# Polyphenolic Compounds from Lespedeza Bicolor Root Bark Inhibit Progression of Human Prostate Cancer Cells via Induction of Apoptosis and Cell Cycle Arrest

**DOI:** 10.3390/biom10030451

**Published:** 2020-03-14

**Authors:** Sergey A. Dyshlovoy, Darya Tarbeeva, Sergey Fedoreyev, Tobias Busenbender, Moritz Kaune, Marina Veselova, Anatoliy Kalinovskiy, Jessica Hauschild, Valeria Grigorchuk, Natalya Kim, Carsten Bokemeyer, Markus Graefen, Petr Gorovoy, Gunhild von Amsberg

**Affiliations:** 1 Department of Oncology, Hematology and Bone Marrow Transplantation with Section Pneumology, Hubertus Wald-Tumorzentrum, University Medical Center Hamburg-Eppendorf, 20251 Hamburg, Germany; tobias.busenbender@gmx.de (T.B.); moritz.kaune@stud.uke.uni-hamburg.de (M.K.); j.hauschild@uke.de (J.H.); c.bokemeyer@uke.de (C.B.); g.von-amsberg@uke.de (G.v.A.); 2 Laboratory of Biologicaly Active Compounds, School of Natural Sciences, Far Eastern Federal University, Vladivostok 690091, Russia; 3 Martini-Klinik, Prostate Cancer Center, University Hospital Hamburg-Eppendorf, 20251 Hamburg, Germany; graefen@martini-klinik.de; 4 G.B. Elyakov Pacific Institute of Bioorganic Chemistry, Far-East Branch, Russian Academy of Sciences, Vladivostok 690022, Russia; tarbeeva1988@mail.ru (D.T.); fedoreev-s@mail.ru (S.F.); veselmv@mail.ru (M.V.); kaaniv@piboc.dvo.ru (A.K.); natalya_kim@mail.ru (N.K.); petrgorovoy@gmail.com (P.G.); 5 Federal Scientific Center of the East Asia Terrestrial Biodiversity (Institute of Biology and Soil Science), Far Eastern Branch, Russian Academy of Sciences, Vladivostok 690022, Russia; kera1313@mail.ru

**Keywords:** Polyphenolic compounds, *Lespedeza bicolor*, pterocarpans, prostate cancer, apoptosis, cell cycle

## Abstract

From a root bark of *Lespedeza bicolor* Turch we isolated two new (7 and 8) and six previously known compounds (1–6) belonging to the group of prenylated polyphenols. Their structures were elucidated using mass spectrometry, nuclear magnetic resonance and circular dichroism spectroscopy. These natural compounds selectively inhibited human drug-resistant prostate cancer in vitro. Prenylated pterocarpans 1–3 prevented the cell cycle progression of human cancer cells in S-phase. This was accompanied by a reduced expression of mRNA corresponding to several human cyclin-dependent kinases (CDKs). In contrast, compounds 4–8 induced a G1-phase cell cycle arrest without any pronounced effect on CDKs mRNA expression. Interestingly, a non-substituted hydroxy group at C-8 of ring D of the pterocarpan skeleton of compounds 1–3 seems to be important for the CDKs inhibitory activity.

## 1. Introduction

*Lespedeza bicolor* Turcz. is a shrub plant belonging to the Leguminosae family. It is widely distributed in East Asia, including the Primorye region of the Russian Far East [1]. In traditional folk medicine, this plant is used for the treatment of nephritis, azotemia, inflammation, hyperpigmentation, energy depletion, diabetes and diuresis [2,3,4,5,6]. In addition, *L. bicolor* extracts possess antioxidant, anti-tyrosinase, anti-inflammatory, estrogenic, antimicrobial and antifungal activities [6,7,8].

Recently, a group from Korea has shown that *L. bicolor* extracts exert a potent memory-enhancing effect when treating cognitive dysfunction induced by amyloid β peptide (25–35) in mice models [9]. Additionally, this extract has been described as a promising therapeutic tool to prevent diabetic nephropathy in methylglyoxal (MGO)-induced models both in vitro and in vivo [10]. Remarkably, the *L. bicolor* extract reduced hyperglycemia-induced hepatic damage, hepatic oxidative stress, and inflammation, as well as liver fibrosis [11]. Finally, this extract inhibited the growth of lung carcinoma LU-1 and prostate cancer LNCaP cells [12]. However, only a little information is available on the compounds responsible for the biological activity of the extract and even less is known about mechanisms of action of these substances.

We have previously isolated several polyphenolic compounds from *L. bicolor* stem bark, which were able to inhibit the growth of human cancer HTB-19, Kyse-30, and HEPG-2 cells [13]. First insights into the mechanism of action were reported for pterocarpans, coumestans, and arylbenzofurans recently isolated from *L. bicolor.* These natural compounds were found to promote cell death via induction of a G1 cell cycle arrest, reduction of Bcl-2 levels, and induction of PARP cleavage in Jurkat blood cancer cells [14]. However, to date no significant reports on the mechanisms of action of the purified *L. bicolor* metabolites are available.

In the current study, we further investigated the metabolites of *L. bicolor* root bark collected in the Primorye Region (Russian Federation). Therefore, we isolated several new as well as previously known prenylated polyphenolic compounds, investigated their cytotoxic properties and the mechanism of action in human drug-resistant prostate cancer cells.

## 2. Materials and Methods

### 2.1. General Experimental Procedures

Optical rotations were measured on a PerkinElmer 343 polarimeter (Perkin Elmer, Waltham, MA, USA). The UV spectra were obtained using a UV-1601 PC spectrophotometer (Shimadzu, Kyoto, Japan). The CD spectra were obtained on a Chirascan-plus Quick Start CD Spectrometer (Applied Photophysics Limited, Leatherhead, UK) (acetonitrile, 20 °C). The IR spectrum was recorded on a Vector 22 fourier-transform infrared spectrometer spectrophotometer (Bruker, Rheinstetten, Germany). The ^1^H, ^13^C, and two-dimensional (2D) NMR spectra were recorded in CDCl_3_ at 30 °C using NMR Bruker AVANCE III DRX-700 and DRX-500 instruments (Bruker, Karlsruhe, Germany). The chemical shift values (*δ*) and the coupling constants (*J*) are given in parts per million and Hz, respectively.

### 2.2. Plant Material

*L. bicolor* was collected in Khasansky District (Andreevka village) of the Primorye Region (The Russian Federation) from a grassy dry meadow in August 2016 by academician P.G. Grovoy. Voucher specimen No. 103608 is preserved in the herbarium of the Laboratory of Chemotaxonomy (G.B. Elyakov Pacific Institute of Bioorganic Chemistry, FEB RAS).

### 2.3. Analytical and Preparative HPLC

The analytical HPLC was carried out using an Agilent Technologies 1100 series HPLC system (Agilent Technologies, Waldbronn, Germany) equipped with a VWD detector (λ = 280 nm). The extracts were analyzed using a Supelco Analytical HS-C18 (Supelco Analytical, Bellefonte, PA, USA) column (3 μm, 4.6, 75 mm) thermostated at 30 °C. The mobile phase consisted of 1% aqueous acetic acid (A) and acetonitrile containing 1% acetic acid (B). For the analysis, the following gradient steps were programmed: 0–2 min—5% B, 2–4 min—5–20% B, 5–17 min—20–50% B, 18–23 min—50–90% B, 24–25 min—90–100% B, 16–27 min—100% B, 28–33 min—100–5% B. The flow rate was 0.8 mL/min. The data were analyzed with the ChemStation software (v. 09, Agilent Technologies, Waldbronn, Germany).

The preparative HPLC was carried out using a Shimadzu HPLC system equipped with an LC-20AT pump and SPD-20A detector (λ = 280 nm) (Shimadzu, Kyoto, Japan). The polyphenolic compounds were purified using a Supelco Analytical HS-C18 (Supelco Analytical, Bellefonte, PA, USA) column (3 μm, 4.6, 75 mm). The mobile phase consisted of 1% aqueous acetic acid (A) and acetonitrile containing 1% acetic acid (B). For the analysis, the following gradient steps were programmed: 0–2 min—5–10% B, 2–4 min—10–20% B, 4–7 min—20–30% B, 7–10 min—30–40% B, 10–12 min—40–50% B, 12–17 min—50–100% B, 17–25 min—100–5% B. The flow rate was 1 mL/min.

The data were analyzed with the LabSolutions software (v. 1.0, Shimadzu, Kyoto, Japan).

### 2.4. HR-ESI-MS

HR-ESI-MS experiments were carried out using a Shimadzu hybrid ion trap–time of flight mass spectrometer (Shimadzu, Kyoto, Japan). The operating settings of the instrument were as follows: electrospray ionization (ESI) source potential, −3.8 and 4.5 kV for negative and positive polarity ionization, respectively, drying gas (N_2_) pressure—200 kPa, nebulizer gas (N_2_) flow—1.5 L/min, temperature for the curved desolvation line (CDL) and heat block—200 °C, detector voltage—1.5 kV and the range of detection—100–900 m/z. The mass accuracy was below 4 ppm. The data were acquired and processed using Shimadzu LCMS Solution software (v.3.60.361, Shimadzu, Kyoto, Japan).

### 2.5. Extraction and Isolation

Air-dried root bark of *L. bicolor* (150 g) was extracted twice under reflux with a CHCl_3_–EtOH mixture at a volume ratio of 3:1 for 3 h (60 °C). The obtained extract of *L. bicolor* root bark (1.7 g) was chromatographed on a polyamide column (80 g, 50–160 µm, Sigma-Aldrich, St. Louis, MI, USA). The column was eluted with a hexane–CHCl_3_ solution system with gradually increasing CHCl_3_ amounts (hexane/CHCl_3_, 1:0, 100:1, 50:1, 40:1) to give fractions 1–13 and then with a CHCl_3_–EtOH solution system with gradually increasing EtOH amounts (CHCl_3_/EtOH, 1:0, 100:1, 50:1, 40:1, *v*/*v*) to give fractions 14–20. The fractions containing polyphenolic compounds according to TLC HPLC data were subjected to further purification. TLC plates were treated with a 3% solution of FeCl_3_ in ethanol to reveal the presence of polyphenolic compounds. Fraction 17 (287 mg) eluted with CHCl_3_–EtOH (50:1) was chromatographed twice over a silica gel column (40–63 µm, Sigma-Aldrich, St. Louis, MI, USA). The column was eluted with a benzene–ethylacetate solution system with gradually increasing ethylacetate amounts (benzene/EtOAc, 1:0, 200:1, 100:1, 50:1, 40:1, *v*/*v*) to obtain compound 1 (10.7 mg). Fraction 18 (176 mg), eluted with CHCl_3_–EtOH (40:1), was also chromatographed on a silica gel column using the same solution system to obtain compounds 1 (5.5 mg), 2 (9.2 mg). Fraction 20 (281 mg), eluted with CHCl_3_ – EtOH (20:1), was also chromatographed on a silica gel column using the same solution system to obtain compounds 6 (15.0 mg) and 8 (2.5 mg).

Fraction 14 (82 mg), eluted with CHCl_3_, was subsequently chromatographed over a silica gel column (40–63 µm) eluted twice with a benzene–ethylacetate solution system with gradually increasing ethylacetate amounts (benzene/EtOAc, 1:0, 200:1, 100:1, 50:1, 40:1, *v*/*v*) resulting in compound 3 (6.6 mg). Fraction 10 (67 mg), eluted with CHCl_3_, was subsequently chromatographed over a silica gel (40–63 µm) column twice using the same solution system to afford compounds 4 (6.1 mg), 5 (8.5 mg), and 7 (3.5 mg).

#### 2.5.1. (6aR,11aR)-8-O-Methyl-6a,11a-Dihydrolespedezol A_2_ (**7**)

White, amorphous powder; [α]_D_^24^ − 201°; (1 mg/mL, MeOH); UV (MeOH), λ_max_ 206, 288, 333 nm; CD (1.89 × 10^−4^ M, CH_3_CN) λ_max_ (Δε) 192 (+80899), 206 (–12011), 216 (–109803), 234 (–59308), 292 (+19668); ^1^H and ^13^C NMR data, see Table 1; HR-ESI-MS *m/z* 421.2007 [M-H]^−^ (calcd for [C_26_H_29_O_5_]^−^ 421.2020,), *m/z* 423.2157 [M+H]^+^ (calcd for [C_26_H_31_O_5_]^+^ 423.2166).

#### 2.5.2. Lespebicolin A (**8**)

Yellow, amorphous powder; [α]_D_^24^ − 95°; (1 mg/mL, MeOH); UV (MeOH), λ_max_ 204, 269, 311 nm; CD (2.46 × 10^−4^ M, CH_3_CN) λ_max_ (Δε) 198 (+40706), 211 (–37771), 235 (–33085), 257(+1494), 271 (–7833), 294 (–7058), 329 (–10749), 392 (+3822); ^1^H and ^13^C NMR data, see Table 2; HR-ESI-MS *m/z* 811.3488 [M-H]^−^ (calcd for [C_50_H_51_O_10_]^−^ 811.3488,), *m/z* 813.3634 [M+H]^+^ (calcd for [C_50_H_53_O_10_]^+^ 813.3633).

### 2.6. Cell Lines and Conditions

The human prostate cancer cell lines PC-3 [docetaxel-resistant, androgen-independent, AR-FL(-), AR-V7(-)], 22Rv1 [docetaxel-sensitive, androgen-independent, AR-FL(+), AR-V7(+)], and the human non-cancer fibroblast cell line MRC-9 were used. PC-3 and 22Rv1 were purchased from ATCC (Manassas, VA, USA). MRC-9 cells were kindly donated by Prof. Dr. med. Sonja Loges (University Medical Center Hamburg-Eppendorf, Hamburg, Germany). Cells were cultured according to the manufacturers’ protocol in 10% FBS/RPMI (PC-3 and 22Rv1) or 10% FBS/DMEM (MRC-9) medium.

### 2.7. MTT Assay

In vitro drug sensitivity MTT assay was performed as previously described [15]. In total, 6000 cells/well were seeded in 96-well plates, incubated overnight, and treated with the investigated drugs for 48 h.

### 2.8. Flow Cytometry Analysis

The effect of the compounds on apoptosis induction and cell cycle progression was analyzed by flow cytometry technique using PI staining as reported before [15]. Cells (0.2 × 10^6^ cells/well) were seeded in six-well plates, incubated overnight, and treated with the investigated drugs for 48 h. The cells were further harvested, fixed with 70% EtOH/H_2_O, stained with propidium iodide (PI), and analyzed using a FACS Calibur machine (BD Bioscience, San Jose, CA, USA). The results were quantified using BD Bioscience Cell Quest Pro software (v.5.2.1., BD Bioscience).

### 2.9. Quantitative Real-Time PCR (qPCR)

The CDKs gene expression was measured using qPCR technique. PC-3 cells were seeded in Petri dishes (1 × 10^6^ cells per ø 6 cm dish in 5 mL) in standard culture media, incubated overnight and treated with the investigated drugs in the fresh culture media for 24 h. Cells were harvested using the cell scraper, washed with PBS and homogenized using QIAshredder (QIAGEN, Hilden, Germany). The total RNA was isolated using PureLink^®^ RNA Mini Kit (Invitrogen, Carlsbad, CA, USA) and the on-column DNA digestion using PureLink™ DNase (Invitrogen). Correspondent RNA (2 µg in 30 µL) and was transcribed into cDNA using Maxima First Strand cDNA Synthesis Kit for RT-qPCR (Thermo Scientific, Vilnius, Lithuania). The following qPCR was performed using 2X KAPA SYBR FAST qPCR Master Mix Optimized for Roche LightCycler 480 (KAPA biosystems, Worburn, MA, USA) according to the manufacturer’s protocol. In total, 20 ng of the template cDNA and 2 pmol of primers were used for each reaction. The PCR conditions were 30 s at 95 °C, followed by 40 cycles of 15 s at 95 °C, 5 s at melting temperature (Tm), and 26 s at 72 °C (fluorescence measurement). Melting curve analysis was performed under the following conditions directly after the PCR run: 10 s at 95 °C, 60 s at 65 °C and 1 s at 97 °C. Relative gene expression was calculated using the 2^-∆∆CT^ method. The expression of CDKs was normalized to GAPDH gene expression. The analysis was performed using primers, purchased from Eurofins MWG-Biotech AG (Ebersberg, Germany). Primers sequences and melting temperatures (Tm) is presented in Table 3.

## 3. Results and Discussion

### 3.1. Isolation and Structure Elucidation of Compounds ***1***–***8***

**Figure 1:**
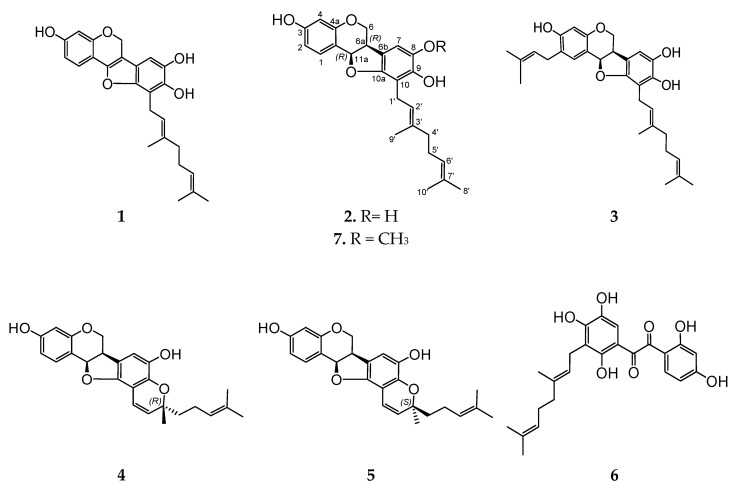

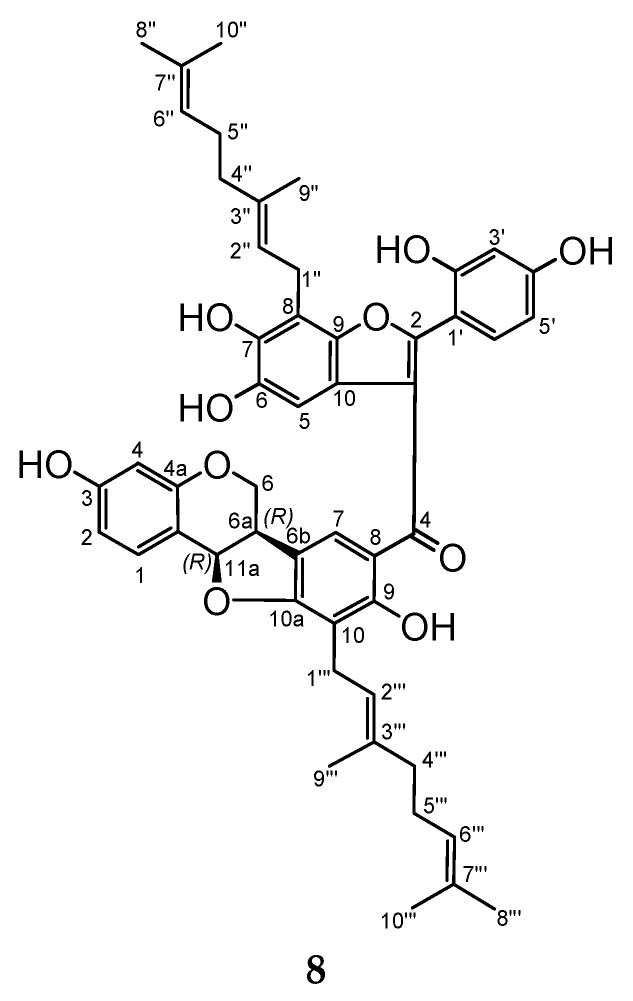
The structures of polyphenolic compounds **1**–**8** isolated from *L. bicolor* root bark and stem bark.

We used a column chromatography on polyamide sorbent to isolate the fractions of the bioactive prenylated polyphenolic compounds. The fractions were tested for the presence of polyphenolics using TLC plates treated with FeCl_3_ and an HPLC–PDA–MS technique. The fractions evaluated positively in a FeCl_3_ test were subsequently separated using silica gel column. Individual compounds were further purified using preparative HPLC. Compounds 1–6 were identified by comparison of their HPLC-PDA-MS and NMR spectra with previously published data [13]. Apart from prenylated polyphenolic compounds 1–6, we isolated and identified two new prenylated pterocarpan derivatives 7 and 8 (Figure 1).

The structures of the isolated compounds were elucidated using mass spectrometry, NMR and CD spectroscopy (for the detailed experimental spectral data of the new compounds please see Supplementary data). Optically active prenylated polyphenolic compound **7** was isolated from *L. bicolor* root bark as a white amorphous powder. Its molecular formula was determined as C_26_H_30_O_5_ based on HRESI-MS data. We observed the [M-H]^−^ ion at *m/z* 421.2007 (calc. for [C_26_H_29_O_5_]^−^ 421.2020) in the negative ion mode, whereas the spectrum in the positive ion mode revealed the presence of the [M+H]^+^ ion at *m/z* 423.2157 (calc. 423.2166 for [C_26_H_31_O_5_]^−^). The ^13^C NMR spectrum contained 26 carbon atoms: 15 carbon atoms of the pterocarpan skeleton, 10 carbon atoms of the geranyl side chain and one carbon atom of the methoxy group (Table 1). ^1^H NMR spectrum also revealed characteristic signals of protons of the pterocarpan skeleton at *δ*_H_ 3.48 (1H, m), 3.66 (1H, t, *J* = 11.0), 4.22 (1H, dd, *J* = 5.0, 11.0) and 5.40 (1H, d, *J* = 7.0) assigned to H-6a, two H-6, and H-11a protons, respectively. The signals at *δ*_H_ 7.40 (1H, d, *J* = 8.4), 6.55 (1H, dd, *J* = 2.6, 8.4), and 6.40 (1H, d, *J* = 2.6) belonged to H-1, H-2, and H-4 protons of ring A, respectively, and formed a spin system. The singlet signal at *δ*_H_ 6.66 (1H, s) was assigned to the aromatic proton at C-7 of ring D. The location of the geranyl side chain at C-10 of the pterocarpan skeleton was determined based on the correlation between the proton signal of 2H-1’ at *δ*_H_ 3.32 and carbon signals of C-9, C-10 и C-10a at *δ*_C_ 144.5, 111.8, and 152.4, respectively, in the HMBC spectrum of **7**. The position of the methoxy group was determined as C-8 based on the observed correlation fbetween the signal of its protons at *δ*_H_ 3.85 and the signal of C-8 at *δ*_C_ 141.0 in the HMBC spectrum of **7** (Table 1). The signals in the ^1^H and ^13^C spectra were completely assigned using HSQC, ROESY, and HMBC data (Table 1). Negative optical rotation value ([α]_D_^24^ − 201º) [16], as well as characteristic Cotton effects in the CD spectrum of **7** (positive bands at λ 192 nm and 292 nm, and negative bands at 216 and 234 nm), confirmed the 6a*R*,11a*R* configuration of the asymmetric centers in **7** [2,17]. Thus, the structure of **7** was determined as (6a*R*,11a*R*)-8-*O*-methyl-6a,11a-dihydrolespedezol A_2_.

Compound **8** was obtained as a yellow amorphous powder. Its HR-ESI-MS spectrum contained the [M-H]^−^ ion at *m/z* 811.3469 in the negative ion mode (calc. 811.3488 for [C_50_H_51_O_10_]^−^) and the [M+H]^+^ ion at *m/z* 813.3634 (calc. 813.3633 for [C_50_H_53_O_10_]^+^) in the positive ion mode. Thus, the molecular formula of **8** was determined as C_50_H_52_O_10_. Both positive and negative MS/MS spectra revealed the intensive signals of the ions [M+H-H_2_O]^+^ at *m/z* 795.3521 (calc. 795.3528) and 793.3360 [M-H-H_2_O]^−^ (calc. 793.3382) due to water molecule loss (Figures S11 and S13, Supplementary data). The presence of the pterocarpan and 2-arylbenzofuran fragments, linked with the carbonyl group, was confirmed using the tandem mass spectrometry technique. The negative MS/MS product ions at *m/z* 419.1476 with the composition [C_25_H_23_O_6_]^−^ (сalc. 419.1500) and at *m/z* 393.1663 with the composition [C_24_H_25_O_5_]^−^ (calc. 393.1707) resulted from the carbonyl bridge degradation and indicated a neutral loss of a pterocarpan fragment (Figures S11 and S12, Supplementary data). The signals at *m/z* 419.1870 (calc. 419.1853) and *m/z* 377.1711 (calc. 377.1747) corresponded to the ions [C_26_H_27_O_5_]^+^ and [C_24_H_25_O_4_]^−^, respectively, and indicated the loss of a neutral pterocarpan fragment. The ^13^C NMR spectrum revealed the presence of 50 carbon atoms: 15 atoms constituted the pterocarpan skeleton, 14 carbon atoms formed the 2-arylbezofuran fragment, 20 atoms belonged to the two geranyl side chains, and one carbon atom belonged to the carbonyl group. The presence of the pterocarpan fragment in **8** was confirmed by the proton signals at *δ*_H_ 3.46 (1H, m), 3.56 (1H, m), 4.01 (1H, m), and 5.58 (1H, d, *J* = 7.3) assigned to H-6a, two H-6, and H-11a protons, respectively. The chemical shift values of these signals were very similar to the signals of H-6a, two H-6, and H-11a protons in the ^1^H spectrum of compounds **1**–**5** and **7**. The geranyl side chain was attached to C-10 of the pterocarpan moiety, which was confirmed by the presence of the HMBC-correlation between the signal of 2H-1‴ at *δ*_H_ 3.32 and the carbon signals of C-9, C-10 и C-10a at *δ*_C_ 164.8, 112.3, and 164.8, respectively.

In the ^1^H spectrum of **8,** we have observed a singlet signal at *δ*_H_ 6.82 assigned to the aromatic proton H-5 and the signals of the ABX proton system in the 2-arylbezofuran fragment at *δ*_H_ 6.50 (1H, d, *J* = 2.3), 6.41 (1H, dd, *J* = 2.3, 8.5), and 7.26 (1H, d, *J* = 8.5) of H-3’, H-5’, and H-6’ protons, respectively. The second geranyl side chain was located at C-8 of the 2-arylbezofuran fragment, which was demonstrated by the presence of the HMBC-correlation between the signals of 2H-1″ at *δ*_H_ 3.69 (2H, d, *J* = 7.3) and the signals of C-7, C-8 and C-9 at *δ*_c_ 147.3, 111.0, and 141.9, respectively. The chemical shift values of the other atoms of 2-arylbezofuran fragment in ^1^H and ^13^C NMR spectra were similar to those for lespecyrtin H_2_ [18].

We observed a correlation between the proton signal of H-7 at *δ*_H_ 7.34 (1H, s) of the pterocarpan fragments and the carbon signal of C-4 of the carbonyl group at *δ*_C_ 194.6 in the HMBC spectrum of **8**. Thus, we concluded that the carbonyl group was attached to C-8 of a pterocarpan fragment. The signal of the hydroxy group proton at C-9 of the pterocarpan fragment had a *δ*_H_ of 12.83, which also confirmed that the carbonyl group was located at C-8 and formed a hydrogen bond with this hydroxy group. The signals in ^1^H and ^13^C NMR spectra of **8** were assigned using HSQC, HMBC, and ROESY experiments. Thus, compound **8**, named lespebicolin A, was assumed to be a dimeric flavonoid consisting of the 2-arylbenzofuran and the pterocarpan fragments linked via carbonyl group.

The absolute configurations of the asymmetric centers at C-6a and C-11a in the pterocarpan fragment of **8** were determined based on the negative optical rotation value ([α]_D_^24^ − 95º) and CD spectral data similar to those of lespecyrtins H_1_–H_4_ [18].

**Figure 2:**
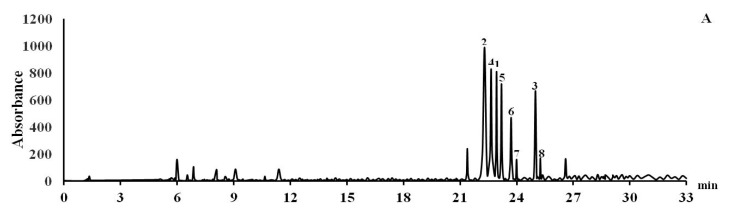

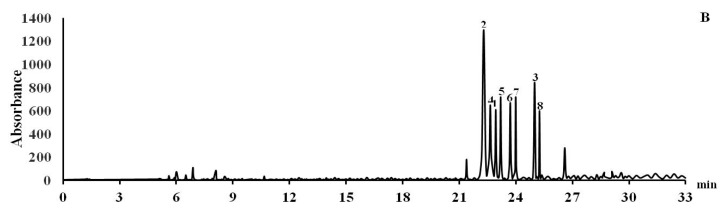
HPLC profiles of *L. bicolor* stem bark (**A**) and root bark (**B**) ethanolic extracts.

Thus, in addition to the six polyphenolic compounds 1–6 previously isolated from *L. bicolor* stem bark [13], we isolated two new pterocarpans 7 and 8 from *L. bicolor* root bark. A comparison of the HPLC profiles of *L. bicolor* stem bark and root bark showed that compounds **7** and **8** are also present in *L. bicolor* stem bark but in smaller amounts than in root bark (Figure 2).

Recently, Korean colleagues isolated five pterocarpans, two new coumestans, and two new arylbenzofurans with prenyl and geranyl substituents in their structures [14]. These isolated compounds contain a methoxy group at C-1 and exhibited antiproliferative effects on human leukemia cells. Of note, we could not find these compounds in the stem bark and root bark of *L. bicolor* harvested in the South of the Primorskiy region of the Russian Far East. The polyphenolic compounds isolated by us from the stem bark and root bark of *L. bicolor* also contained the pterocarpan skeleton and geranyl side chains in their structures. However, they did not have a methoxy group at C-1. Moreover, for the first time, we have isolated a new dimeric flavonoid lespebicolin A (**8**) harboring two geranyl side chains. Previously, the related dimeric flavonoids had been found in *L. homoloba*, *L. floribunda* and *L. cyrtobotria* [18,19]. Thus, the chemical compositions of polyphenolic compounds of *L. bicolor* growing in the Primorskiy region and South Korea differed significantly. This may be because *L. bicolor* samples collected in the Primorye Region (the South of the Russian Far East) and South Korea belong to different variants of this species [20].

### 3.2. Investigation of Anticancer In Vitro Activity of the Compounds ***1***–***8***

Next, we examined the cytotoxic activity of the isolated compounds in prostate cancer cell lines as well as in non-cancer cells. To get a first impression on the impact of these novel compounds for the treatment of advanced prostate cancer, human drug-resistant cell lines PC-3 and 22Rv1 were used. Both cell lines are known to be castration-resistant (androgen-independent). In addition, the cell lines are known to exhibit resistance to novel second-generation androgen receptor (AR) targeting drugs, e.g., abiraterone and enzalutamide, due to the loss or alternative splicing of the AR, respectively [21,22]. Moreover, PC-3 cells have been reported to be rather resistant to docetaxel, and therefore, are known as one of the most aggressive prostate cancer cell lines used as in vitro and in vivo models. Notably, all eight isolated compounds exhibited a cytotoxic activity in both prostate cancer cell lines in the micromolar range, while non-malignant MRC-9 cells were less affected under the treatment (Table 4). The selectivity index evaluation (SI; MRC-9 vs. PC-3 cells) has revealed compounds 1, 2, 3 as well as 6 and 8 to be the most promising and selective in human drug-resistant prostate cancer cells (SI = 2.5 ~ 8) (Table 4). Remarkably, a well-established cytotoxic chemotherapeutic drug cisplatin applied for the treatment of different cancer types and used in the current research as a positive control was less selective (SI = 0.72) (Table 4). For further investigations on the mechanisms of action, we have chosen the aggressive and drug-resistant prostate cancer PC-3 cell line.

**Figure 3:**
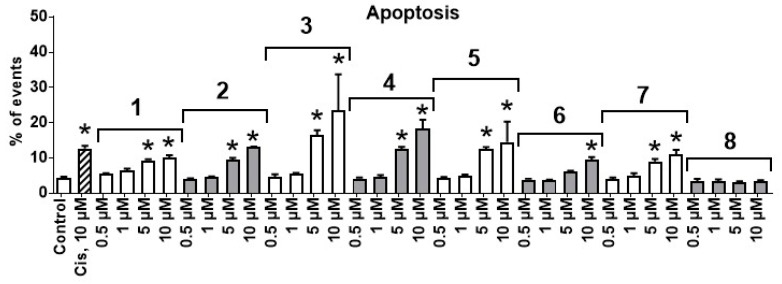
Effect of natural compounds on DNA fragmentation of PC-3 cells. Cells were treated with the investigated compounds for 24 h. The effects were analyzed by flow cytometry. Cells detected as sub-G1 population were considered to be apoptotic. Cisplatin (Cis) was used as a positive control. * *p* ≤ 0.05 (Student’s t-test).

To investigate the mechanism of action contributing to the cytotoxicity in PC-3 cells, we investigated the effects on DNA fragmentation, a well-established marker of cellular apoptosis. Therefore, we evaluated the effects of the isolated compounds at four different concentrations of 0.5 µM, 1 µM, 5 µM, and 10 µM by flow cytometry (Figure 3). All the substances apart from structurally different lespebicolin A (**8**) revealed a significant apoptosis induction at the tested concentrations (Figure 3). Therefore, prenylated dimeric flavonoid **8** may either reveal a non-apoptotic character of cell death or a predominantly antiproliferative rather than cytotoxic effect. Note, compound **3** exhibited the most pronounced apoptosis-inducing effect.

**Figure 4:**
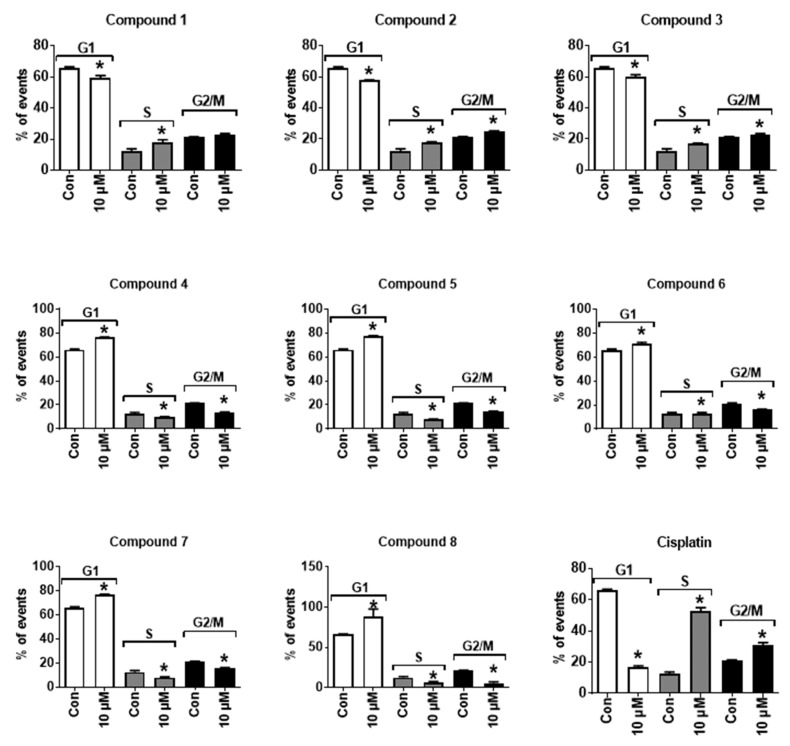
Treatment effects on the progression of the cell cycle of PC-3 cells. Cells were treated with the investigated compounds for 24 h. The effects were analyzed by flow cytometry. Cisplatin was used as a positive control. * *p* ≤ 0.05 (Student’s t-test).

Next, we examined the effects of the isolated compounds on the cell cycle progression of PC-3 cells. For compounds **1**–**3**, a significant accumulation of the cells in S-phase as well as slight accumulation in G2/M-phase was observed (Figure 4). Thus, these compounds mainly induced a S-phase arrest, whereas the compounds **4**–**8** led to a pronounced G1-phase cell cycle arrest (Figure 4).

**Figure 5:**
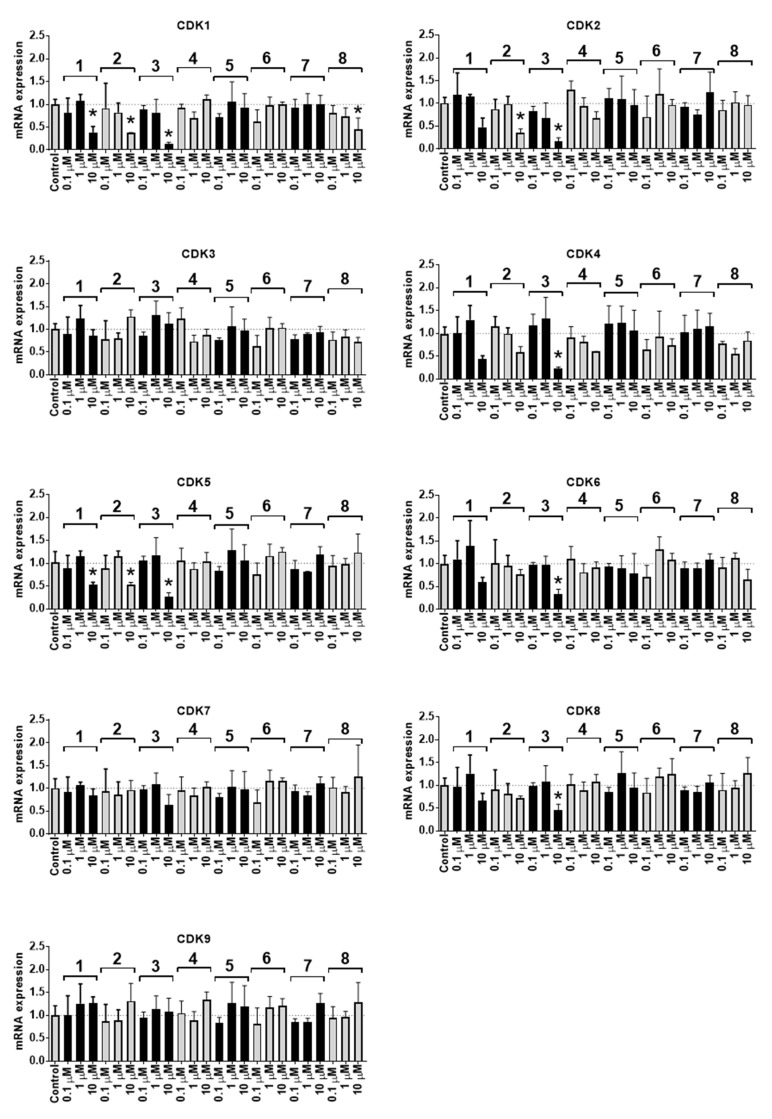
Expression of mRNA correspondent to CDK1-9 in PC-3 cells treated with the isolated compounds **1**–**8** for 24 h. The target gene expression was normalized to GAPDH gene expression. * *p* ≤ 0.05 (Student’s t-test).

Due to the significant alterations of the cell cycle, we further investigated the effects of the isolated compounds on the cyclin-dependent kinases (CDKs). These proteins are known to control proliferation as well as some apoptotic processes. We have examined the expression of mRNAs corresponding to the nine most studied CDKs known to be involved in the cell cycle progression of cancer cells, namely, CDK1–CDK9. Indeed, we detected the inhibitory effects of compounds **1**, **2,** and **3** on the expression of mRNA corresponding to several essential CDKs (Figure 5). The most pronounced effects were observed for CDK1, CDK2, CDK4, and CDK5. Remarkably, these effects correlated well with the cytotoxicity of the isolated compounds. Thus, the strongest inhibitory effect on the corresponding mRNA expression was observed with the most cytotoxic compound **3** (IC_50_ = 2.1 µM in PC-3 cells, Table 3).

CDKs are known to play an important role in the development and progression of several cancer types, including human prostate cancer. These kinases are involved in the cell cycle control and are often overexpressed or mutated in cancer cells [23]. Therefore, CDKs are an attractive target in anticancer therapy and are currently actively explored [23]. In particular, CDK1 and CDK6 are known to phosphorylate AR and activate its transcriptional activity at different phosphorylation sites finally leading to a poorer clinical prognosis [24,25,26]. Expression and activity of CDK2 are associated with prostate cancer relapse in patients and with cancer cell invasion [27]. In addition, the particular importance and clinical relevance of CDK5 for prostate cancer growth have been highlighted in different publications. Thus, it was reported to phosphorylate and stabilize AR leading to the promotion of its transcriptional activity [28,29]. Moreover, CDK5 stimulates the growth of AR-negative prostate cancer cells via Akt activation [30]. Of note, the CDK4/6 inhibitor is currently undergoing clinical trials in metastatic castration-resistant prostate cancer. They can promote cell senescence as well as disruption of cancer cells in vivo by the induction of cytotoxic T cell-mediated cell death. Moreover, inhibition of CDK4/6 may cause S-phase cell cycle arrest, which has been observed for compounds 1–3 (Figure 4) [31,32].

Of note, the other compounds 4–8 did not exhibit pronounced effects on CDKs expression (Figure 5) but still inhibited cell cycle progression, however, in the G1 phase (Figure 4). This effect may result from the other processes related either to apoptosis induction or inhibition of different CDKs at the post-transcriptional (inhibition of CDKs protein expression) or even post-translational levels (inhibition of CDKs activity). Indeed, a free hydroxyl group at C-8 (e.g., pterocarpans 1–3) seems to be important for the inhibition of CDKs mRNA expression by these natural compounds. Hence, compound 2, containing 8-OH was fairly active in this experiment, however, very structurally similar new compound **7** containing a methylated hydroxyl group (8-OCH_3_) did not exhibit this activity; moreover, it was less cytotoxic and had a different effect on cell cycle progression (i.e., G1-phase arrest instead of S-phase arrest). In addition, new compound 8 has a pterocarpan fragment related to compounds 1, 2 and 3. However, this compound is substituted at C-8 and therefore similar to **7** did not exhibit any pronounced effect on CDKs mRNA expression and could not induce an S-phase cell cycle arrest.

## 4. Conclusions

In conclusion, we isolated two new (**7** and **8**) and six recently reported (**1**–**6**) polyphenolic compounds from the root bark of *L. bicolor*. Structures were established using mass spectrometry and NMR spectroscopy. The natural compounds were active and selective in human drug-resistant prostate cancer cells in vitro. Prenylated pterocarpans **1**–**3** effectively inhibited cell cycle progression of human cancer cells in S-phase. This was associated with the inhibition of mRNA expression corresponding to several human CDKs. Compounds **4**–**8** induced G1-phase cell cycle arrest without any pronounced effect on CDK mRNA expression. The non-substituted hydroxy group at C-8 in ring D of the pterocarpan skeleton seems to be important for the CDKs inhibitory activity.

## Figures and Tables

**Table 1:** ^1^H (700 MHz), ^13^C (175 MHz), and HMBC NMR data for compound 7 (*δ* in ppm, *J* in Hz).

N	*δ_H_* (*J* in Hz)	*δ_C_*	HMBC	ROESY
1	7.40 d (8.4), 1H	132.4	C-3, 4, 4a	2
2	6.55dd (2.6, 8.4), 1H	109.5	C-3, 4, 11b	1
3		156.7		
4	6.40 dd (2.6), 1H	103.5	C-2, 3, 4a, 11b	
4a		156.6		
6	4.22 dd (5.0, 11,0), 1H	66.6	C-4a, 6a, 6b, 11a	6, 6a, 7
	3.66 t (11.0), 1H	66.6	C-4a, 6a, 6b, 11a	6
6a	3.48 m, 1H	40.7	C-6, 6b, 10a	6, 11a
6b		115.6		
7	6.66 s, 1H	105.1	C-6, 6a, 6b, 8, 9, 10, 10a	6, OCH_3_-8
8		141.0		
9		144.5		
10		111.8		
10a		152.4		
11a	5.40 d (7.0), 1H	77.4	C-1, 4a, 6, 6a, 6b, 11b	6a
11b		113.3		
1’	3.32 m, 2H	23.0	C-8, 9, 10, 2’, 3’	2’, 9’
2’	5.30 t (7.2), 1H	121.5	C-10, 1’, 4’, 9’	1’, 4’
3’		135.6		
4’	1,96 m, 2H	39.8	C-2’, 3’, 5’, 6’, 9’	2’, 6’
5’	2.04 m (8.3, 7.4, 7.2), 2H	26.7	C-3’, 4’, 6’, 7’	6’
6’	5.07 t (6.8), 1H	124.4	C-5’, 8’, 10’	4’, 5’
7’		131.1		
8’	1.64 s, 3H	25.6	C-6’, 7’, 10’	
9’	1.76 s, 3H	16.1	C-2’, 3’, 4’	1’
10’	1.56 s, 3H	17.6	C-6’, 7’, 8	
OH-3	4.78 bs, 1H			
OCH_3_-8	3.85, 3H	56.9	C-8	7
OH-9	5.69 s, 1H		C-8, 9, 10, 10ª	

**Table 2:** ^1^H (700 MHz), ^13^C (175 MHz) and HMBC NMR data for compound **8** (*δ* in ppm, *J* in Hz).

N	*δ_H_* (*J* in Hz)	*δ_C_*	HMBC
***Upper***			
2		153.7	
3		119.5	
4		194.6	
5	6.82 s, 1H	103.4	C-6, 7, 8, 9, 10
6		141.5	
7		147.3	
8		111.0	
9		141.9	
10		116.9	
1’		110.7	
2’		156.6	
3’	6.50 d (2.3), 1H	105.0	C-1’, 2’, 4’, 5’
4’		158.6	
5’	6.41 dd (2.3, 8.5), 1H	108.6	C-1’, 3’
6’	7.26 d (8.5), 1H	132.2	C-2, 2’, 4’
1″	3.69 d (7.3), 2H	23.4	C-7, 8, 9, 2″, 3″
2″	5.42 t (7.3), 1H	120.4	C-4″, 9″
3″		139.3	
4″	2.11 m, 2H	39.7	C-2″, 3″, 5″, 6″, 9″
5″	2.13 m, 2H	26.4	C-4″, 6″, 7″
6″	5.07 m, 1H	123.8	
7″		132.1	
8″	1.68 s, 3H	25.7	C-6″, 7″, 10″
9″	1.86 s, 3H	16.3	C-2″, 3″, 4″
10″	1.60 s, 3H	17.7	C-6″, 7″, 8″
***Lower***			
1	7.38 d (8.5), 1H	132.4	C-3, 4a,11a
2	6.56 dd (2.4, 8.5), 1H	110.1	C-4, 11b
3		157.2	
4	6.38 d (2.4), 1H	103.6	C-2, 3, 4a, 11b
4a		156.4	
6	4.01 m, 1H	66.3	
	3.56 m, 1H	66.3	
6a	3.46 m, 1H	39.6	
6b		118.4	
7	7.34 s, 1H	127.1	C-4 (upper), 6a, 9, 10a
8		113.4	
9		164.8	
10		112.3	
10a		164.8	
11a	5.58 d (7.3), 1H	79.2	C-1, 4a, 6, 11b
11b		112.5	
1‴	3.32 d (7.2), 2H	22.2	C-9, 10, 10a, 2‴, 3‴
2‴	5.26 t (7.2), 1H	121.0	C-10, 1‴, 4‴, 9‴
3‴		136.0	
4‴	1.98 m, 2H	39.8	C-2‴, 3‴, 5‴, 6‴, 9‴
5‴	2.04 m, 2H	29.7	C-3‴, 4‴, 6‴, 7‴
6‴	5.07 m, 1H	124.4	C-8‴, 10‴
7‴		131.3	
8‴	1.64 s, 3H	25.6	C-6‴, 7‴, 10‴
9‴	1.77 s, 3H	16.2	C-2‴, 3‴, 4‴
10‴	1.56 s, 3H	17.6	C-6‴, 7‴, 8‴
OH-9	12.83, s, 1H		

**Table 3:** The sequences and melting temperatures (Tm) of the primers used for qPCR.

Primer	Sequence	Tm [°C]
CDK1_for	ACAGGTCAAGTGGTAGCCATGA	60
CDK1_rev	ACCTGGAATCCTGCATAAGCA	
CDK2_for	TTCTCATCGGGTCCTCCACC	61
CDK2_rev	TCGGTACCACAGGGTCACCA	
CDK4_for	CTGTGCCACATCCCGAACTG	61
CDK4_rev	GCCTCTTAGAAACTGGCGCA	
CDK5_for	CCACAACATCCCTGGTGAACGT	62
CDK5_rev	CCTCTTCTGCTGAGATACGCTG	
CDK6_for	CCGAAGTCTTGCTCCAGTCC	61
CDK6_rev	GGGAGTCCAATCACGTCCAA	
CDK7_for	TCACATCTTCAGTGCAGCAGG	59
CDK7_rev	TGGCAGCTGACATCCAGGT	
CDK8_for	AGCGGGTCGAGGACCTGTTT	62
CDK8_rev	CATGCCGACATAGAGATCCCAG	
CDK3_for	TCGCTGCTCAAGGAACTGAAGC	62
CDK3_rev	GTCCTGGCTGAGGAACTCAAAC	
CDK9_for	CCATTACAGCCTTGCGGGAGAT	62
CDK9_rev	CAGCAAGGTCATGCTCGCAGAA	

**Table 4:** Inhibition concentrations 50% (IC_50_s) of the investigated compounds in different cell lines. The cells were treated for 48 h. Cisplatin was used as a positive control. Selectivity index (SI) was calculated as [IC_50_ in MRC-9 cells] / [IC_50_ in PC-3 cells].

		IC_50_, µM								
	Compound	**1**	**2**	**3**	**4**	**5**	**6**	**7**	**8**	**Cisplatin**
Cell line	PC-3	6.73	5.24	2.1	16.37	15.23	19.26	14.86	9.24	21.0
	22Rv1	20.14	13.85	6.38	29.53	35.81	46.9	30.51	17.51	4.53
	MRC-9	48.51	37.57	16.82	27.18	24.61	>50	24.4	23.95	15.12
	Selectivity index (MRC-9 vs. PC-3)	7.21	7.17	7.99	1.66	1.62	>2.6	1.64	2.59	0.72

## Supplementary Materials

The following are available online at https://www.mdpi.com/2218-273X/10/3/451/s1, Figures S1–S20.
