# Supplementary material for: Polyphenolic Compounds from Lespedeza Bicolor Root Bark Inhibit Progression of Human Prostate Cancer Cells via Induction of Apoptosis and Cell Cycle Arrest
Source: Biomolecules. 2020 Mar 14;10(3):451. doi: 10.3390/biom10030451 (PMC7175281; doi:10.3390/biom10030451)
Supplement: Supplementary file 1 [file biomolecules-10-00451-s001.pdf]

Supplementary data

Polyphenolic compounds from *Lespedeza bicolor* root bark inhibit progression of human prostate cancer cells via induction of apoptosis and cell cycle arrest

Sergey A. Dyshlovoy[1,2,3,*], Darya V. Tarbeeva[4], Sergey A. Fedoreyev[4], Tobias Busenbender[1], Moritz Kaune[1], Marina V. Veselova[4], Anatoliy I. Kalinovskiy[4], Jessica Hauschild[1], Valeria P. Grigorchuk[5], Natalya Yu. Kim[4], Carsten Bokemeyer[1], Markus Graefen[3], Petr G. Gorovoy[4], Gunhild von Amsberg[1,3]

[1] Department of Oncology, Hematology and Bone Marrow Transplantation with Section Pneumology, Hubertus Wald-Tumorzentrum, University Medical Center Hamburg-Eppendorf, Hamburg, Germany; dyshlovoy@gmail.com (S.A.D.), j.hauschild@uke.de (J.H.), moritz.kaune@stud.uke.uni-hamburg.de (M.K.), tobias.busenbender@gmx.de (T.B.), c.bokemeyer@uke.de (C.B.), g.von-amsberg@uke.de (G.v.A.)

[2] School of Natural Sciences, Far Eastern Federal University, Vladivostok, Russian Federation адрес

[3] Martini-Klinik, Prostate Cancer Center, University Hospital Hamburg-Eppendorf, Hamburg, Germany; graefen@martini-klinik.de (M.G.)

[4] G.B. Elyakov Pacific Institute of Bioorganic Chemistry, Far-East Branch, Russian Academy of Sciences, Vladivostok, Russian Federation; tarbeeva1988@mail.ru (D.V.T.), fedoreev-s@mail.ru (S.A.F.), veselmv@mail.ru (V.M.V.), kaaniv@piboc.dvo.ru (A.I.K.), natalya_kim@mail.ru (N.Yu.K.), petrgorovoy@gmail.com (P.G.G.)

[5] Federal Scientific Center of the East Asia Terrestrial Biodiversity (Institute of Biology and Soil Science), Far Eastern Branch, Russian Academy of Sciences, Prospect 100-let Vladivostoku 159, Vladivostok, 690022, Russia; kera1313@mail.ru (V.P.G.)

* Correspondence: dyshlovoy@gmail.com or s.dyshlovoy@uke.de; Tel.: +49-40-7410-51950

Contents

Fig. 1. – UV mass spectrum of (6a*R*, 11a*R*)-8-methoxy-6a, 11a-dihidrolespedezol A_2 (**7**)..3

Fig. 2.– HR-ESI mass spectrum of (6a*R*, 11a*R*)-8-methoxy-6a, 11a-dihidrolespedezol A_2 (**7**) (positive ion mode) 3

Fig. 3. – HR-ESI mass spectrum of (6a*R*, 11a*R*)-8-methoxy-6a, 11a-dihidrolespedezol A_2 (**7**) (negative ion mode) 3

Fig. 4. –CD spectrum of (6a*R*, 11a*R*)-8-methoxy-6a, 11a-dihidrolespedezol A_2 (**7**)....4

Fig. 5. – ^{1}H spectrum of (6aR, 11aR)-8-methoxy-6a, 11a-dihidrolespedezol A_2 (**7**) 5

Fig. 6. – ^{13}C spectrum of (6a*R*, 11a*R*)-8-methoxy-6a, 11a-dihidrolespedezol A_2 (**7**) 6

Fig. 7. – HMBC spectrum of (6a*R*, 11a*R*)-8-methoxy-6a, 11a-dihidrolespedezol A_2 (**7**) 7

Fig. 8. –UV spectrum of lespebicolin A (**8**)....8

Fig. 9. – HR-ESI mass spectrum of lespebicolin A (**8**) (positive ion mode) 8

Fig. 10. – HR-ESI mass spectrum of lespebicolin A (**8**) (negative ion mode)........................8

Fig. 11. – MS/MS spectrum of the molecular ion at *m/z* 811.3469 of lespebicolin A (**8**) (negative ion mode)........................9

Fig. 12. MS/MS cleavage of the ion $[M-H]^-$ at *m/z* 811.3469 of lespebicolin A (**8**) (negative ion mode)........................9

Fig. 13. MS/MS spectrum of the molecular ion at *m/z* 813.3639 of lespebicolin A (**8**) (positive ion mode)........................10

Fig. 14. MS/MS cleavage of the ion $[M+H]^+$ at *m/z* 813.3639 of lespebicolin A (**8**) (positive ion mode)........................10

Fig. 15. – CD spectrum of lespebicolin A (**8**)........................11

Fig. 16. – ^1H spectrum of lespebicolin A (**8**)........................12

Fig. 17. – ^{13}C spectrum of lespebicolin A (**8**)........................13

Fig. 18. – HMBC spectrum of lespebicolin A (**8**)........................14

Fig. 19. – IR spectrum of lespebicolin A (**8**)........................15

Fig. 20. – IR spectrum of lespebicolin A (**8**)........................16

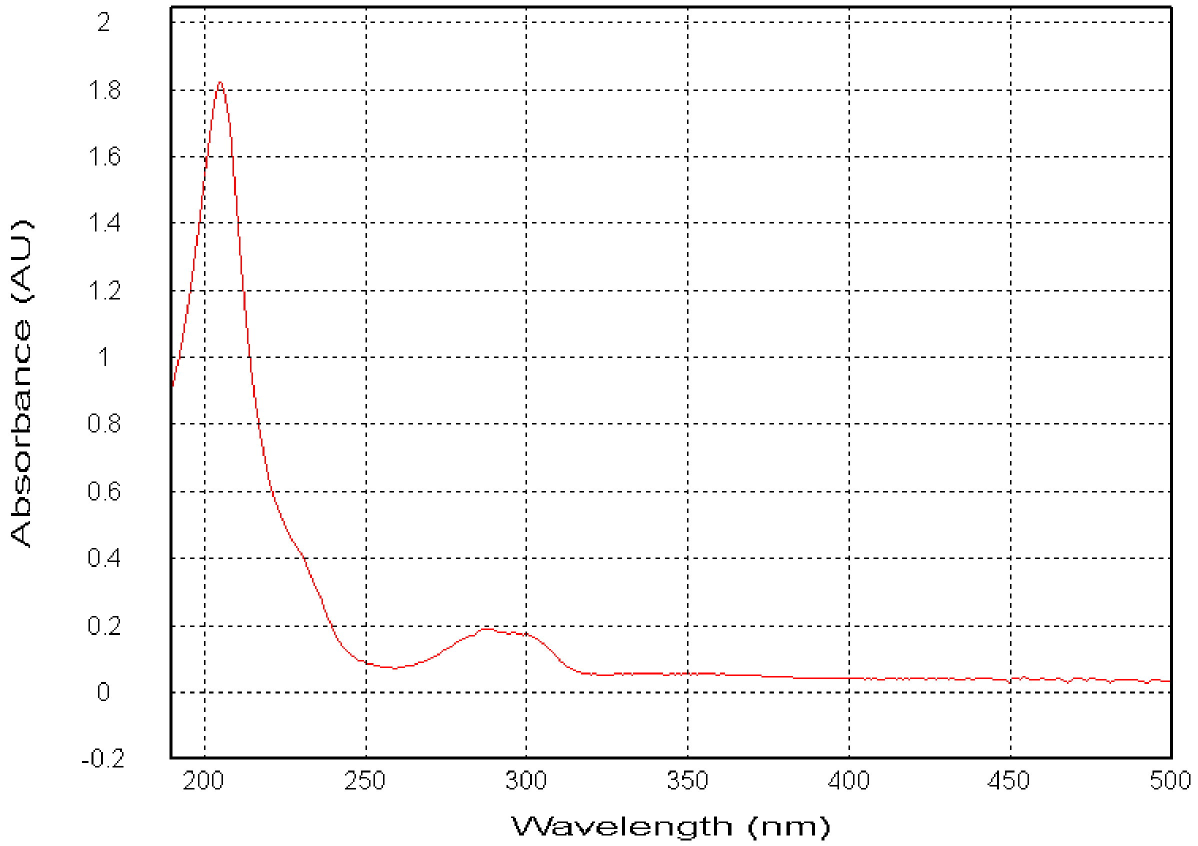

Fig. S1. – UV mass spectrum of (6a*R*, 11a*R*)-8-methoxy-6a, 11a-dihidrolespedezol A_2 (**7**)

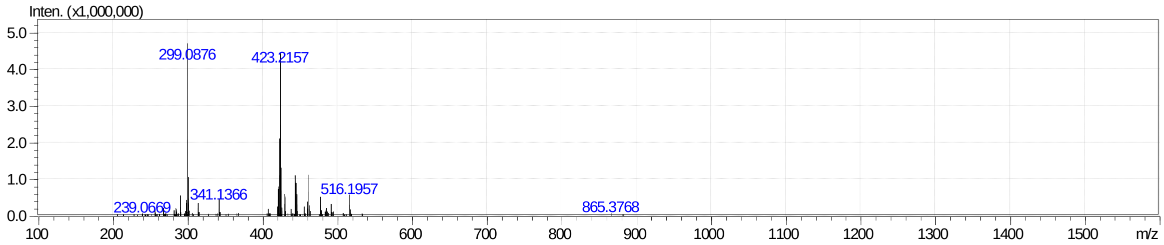

Fig. S2.– HR-ESI mass spectrum of (6a*R*, 11a*R*)-8-methoxy-6a, 11a-dihidrolespedezol A_2 (**7**) (positive ion mode)

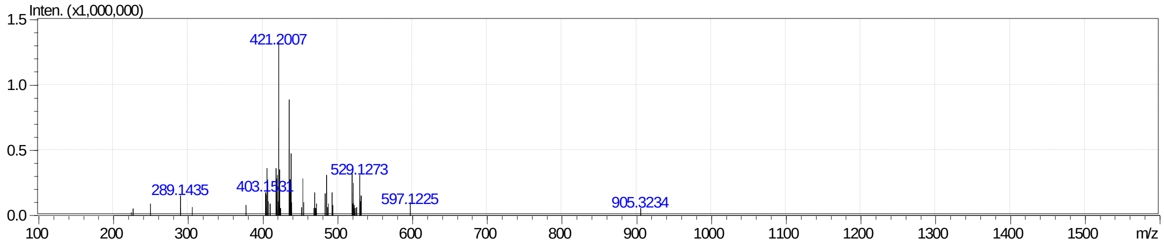

Fig. S3. – HR-ESI mass spectrum of (6a*R*, 11a*R*)-8-methoxy-6a, 11a-dihidrolespedezol A_2 (**7**) (negative ion mode)

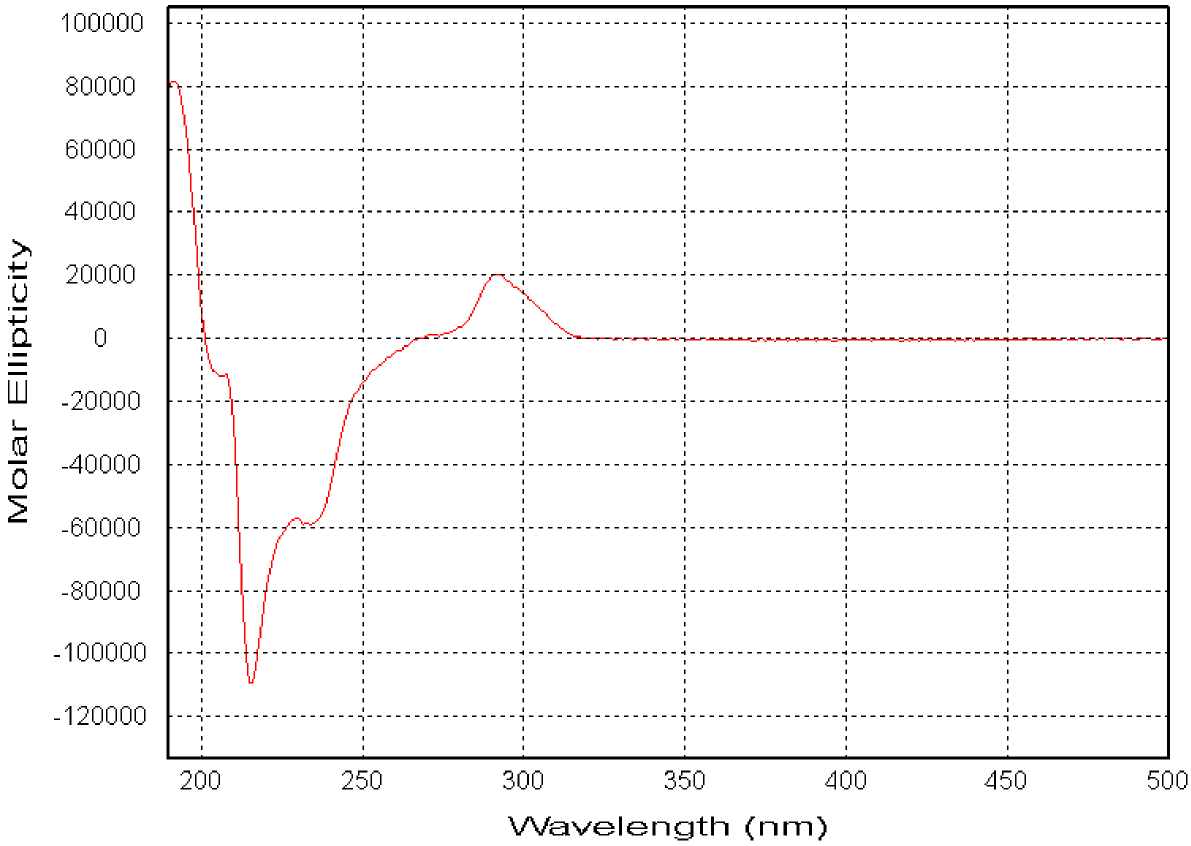

Fig. S4. –CD spectrum of (6a*R*, 11a*R*)-8-methoxy-6a, 11a-dihidrolespedezol A_2 (**7**)

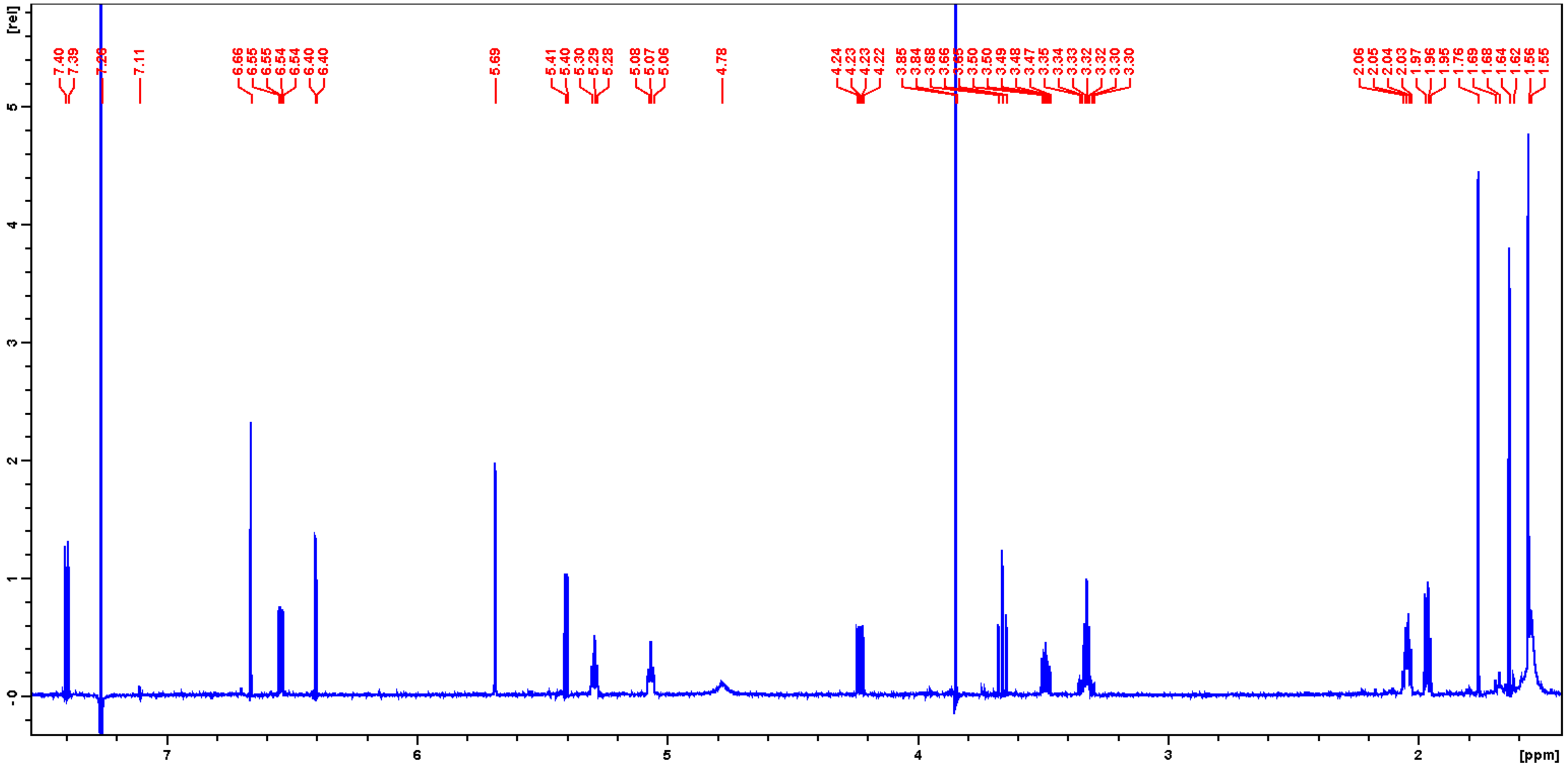

Fig. S5. – ^{1}H spectrum of (6aR, 11aR)-8-methoxy-6a, 11a-dihidrolespedezol A₂ (**7**)

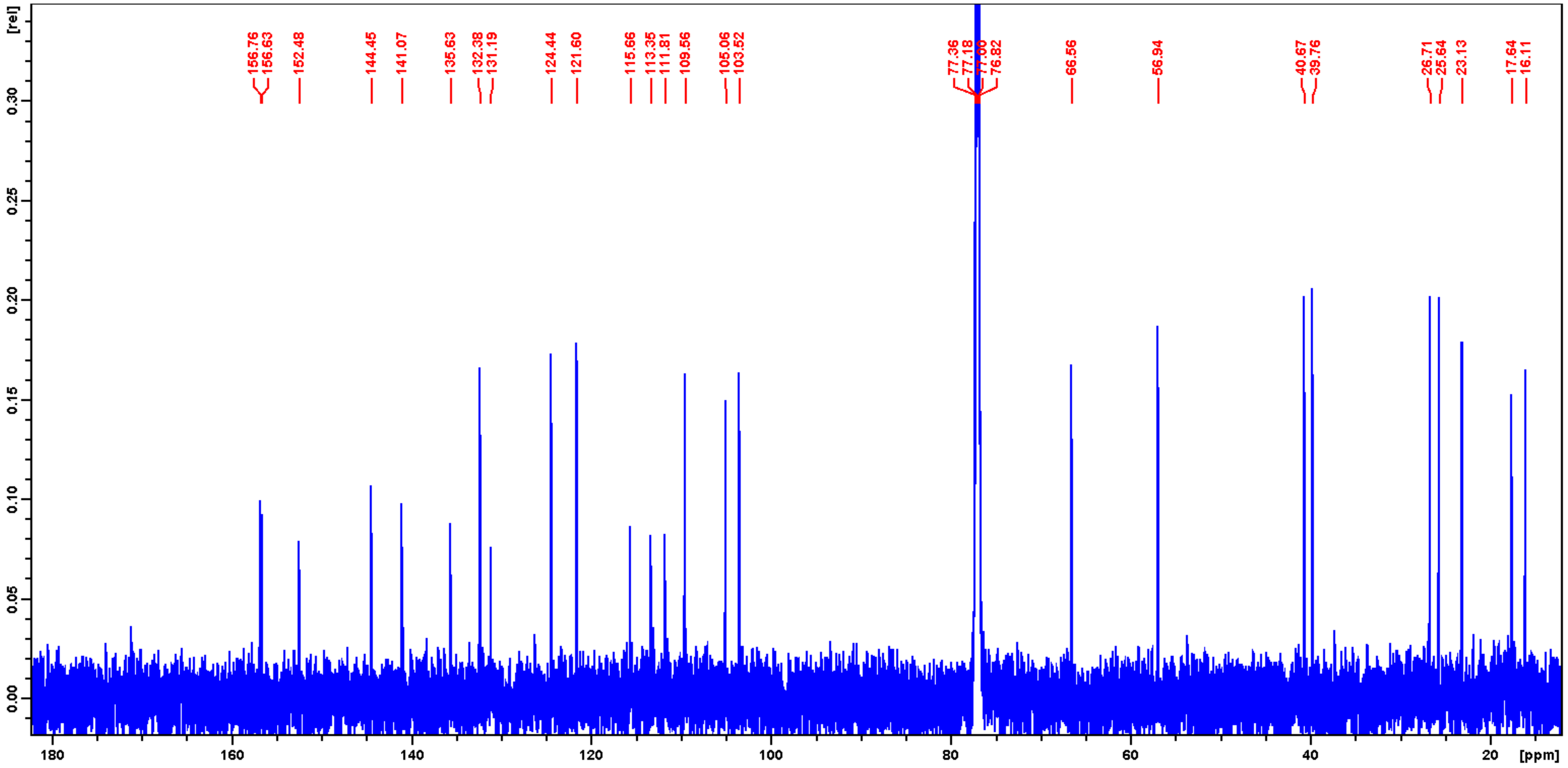

Fig. S6. – ^{13}C spectrum of (6a*R*, 11a*R*)-8-methoxy-6a, 11a-dihidrolespedezol A$_2$ (**7**)

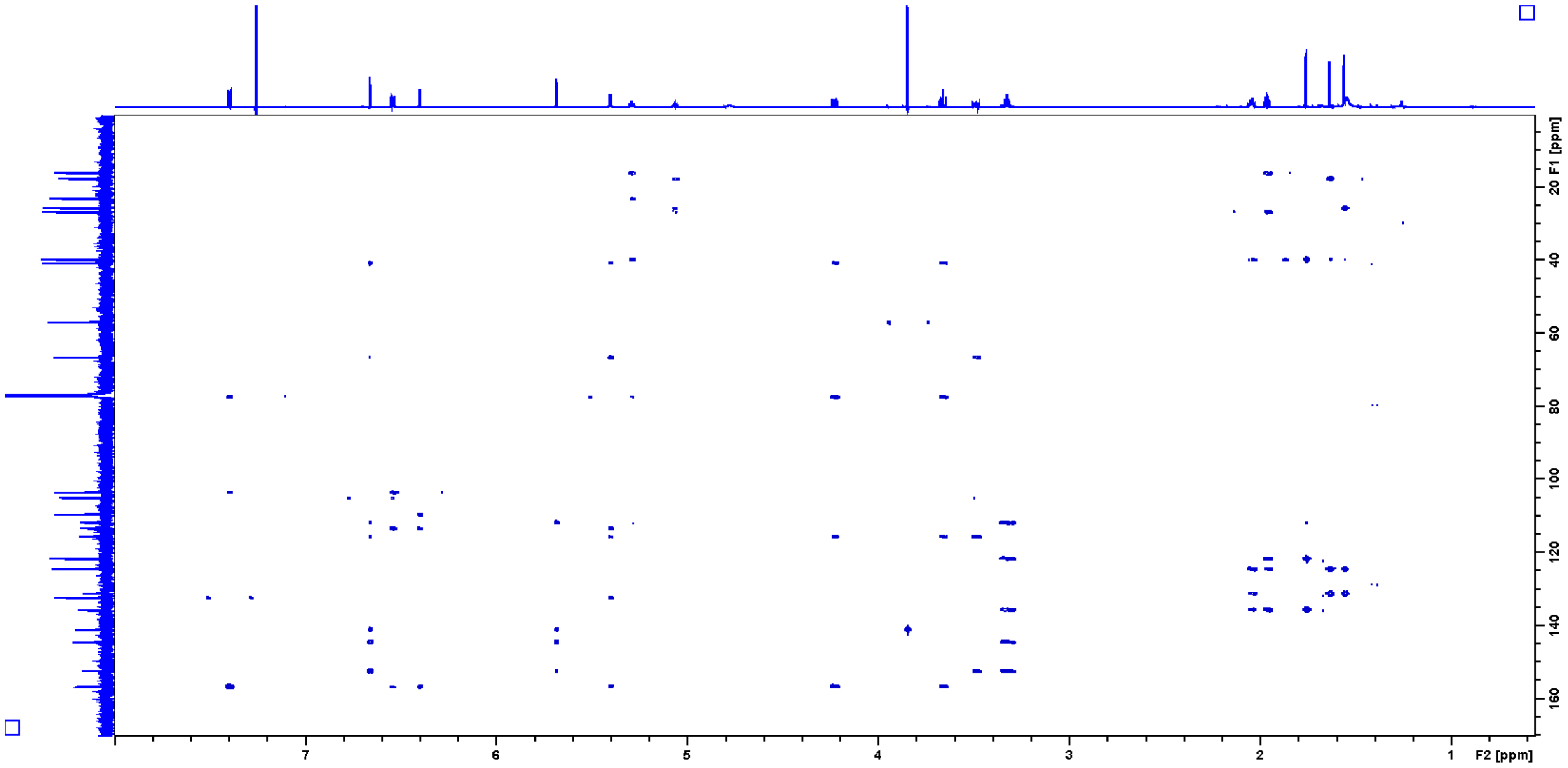

Fig. S7. – HMBC spectrum of (6a*R*, 11a*R*)-8-methoxy-6a, 11a-dihidrolespedezol A2 (**7**)

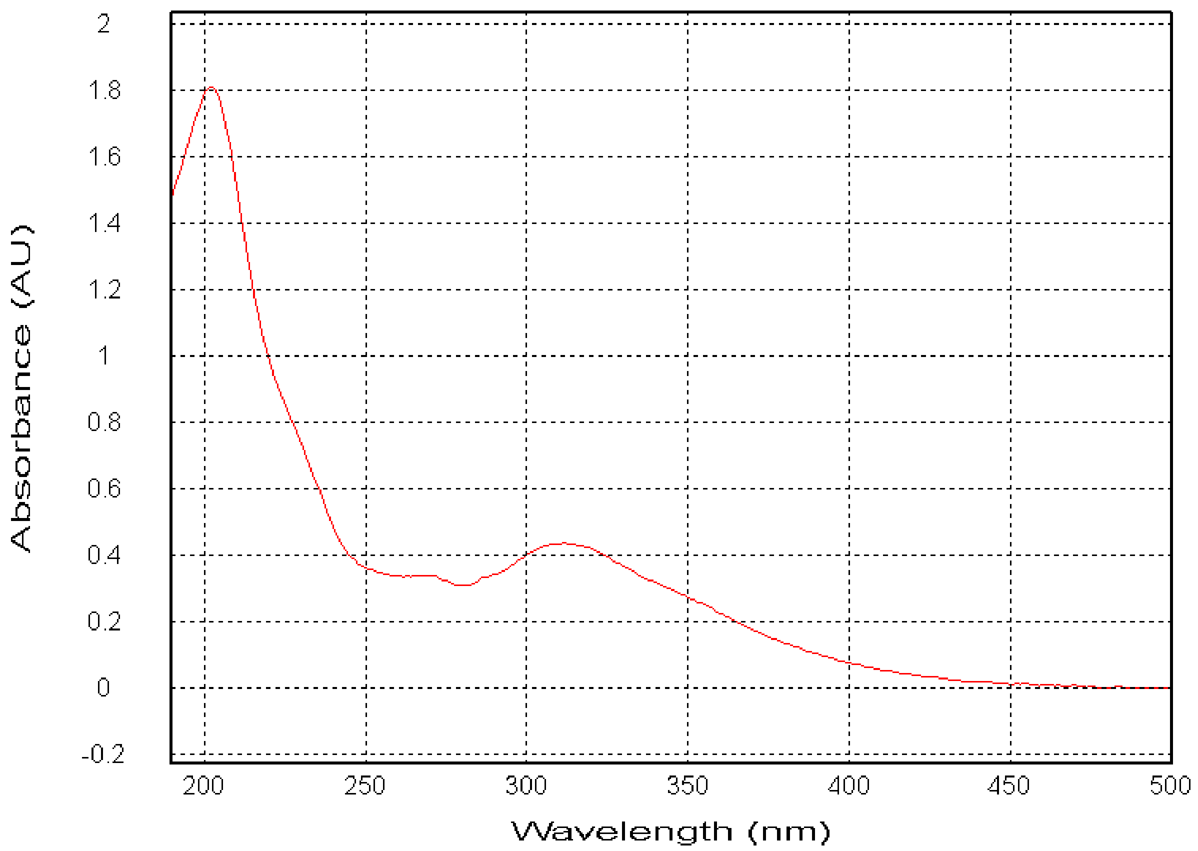

Fig. S8. –UV spectrum of lespebicolin A (**8**)

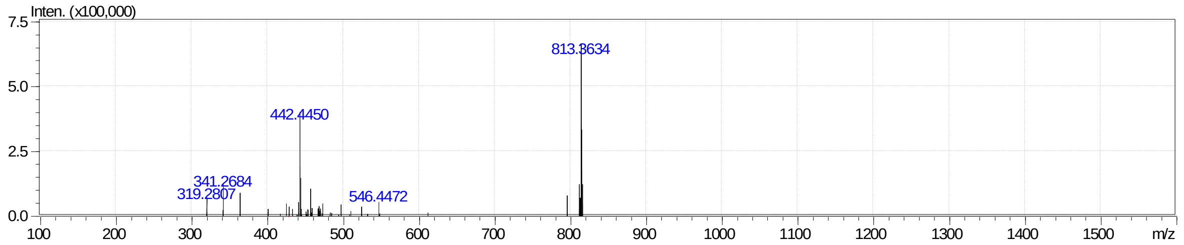

Fig. S9. – HR-ESI mass spectrum of lespebicolin A (**8**) (positive ion mode)

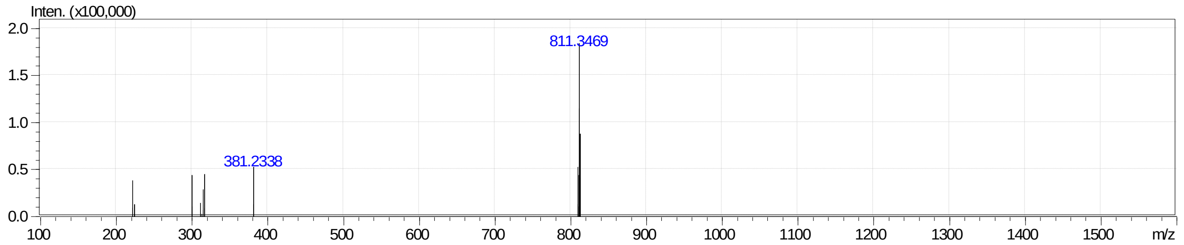

Fig. S10. – HR-ESI mass spectrum of lespebicolin A (**8**) (negative ion mode)

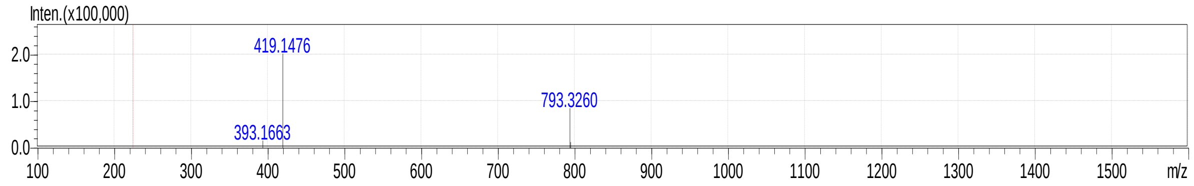

793.3360 – [M-H-H_2O]$^-$ – [$C_{50}H_{49}O_9$]$^-$ (Calculated – 793.3382) – H_2O loss

419.1476 – [M-H-$C_{25}H_{28}O_4$]$^-$ – [$C_{25}H_{23}O_6$]$^-$ (Calculated – 419.1500)

393.1663 – [M-H-$C_{26}H_{26}O_5$]$^-$ – [$C_{24}H_{25}O_5$]$^-$ (Calculated – 393.1707)

Fig. S11. – MS/MS spectrum of the molecular ion at *m/z* 811.3469 of lespebicolin A (**8**) (negative ion mode)

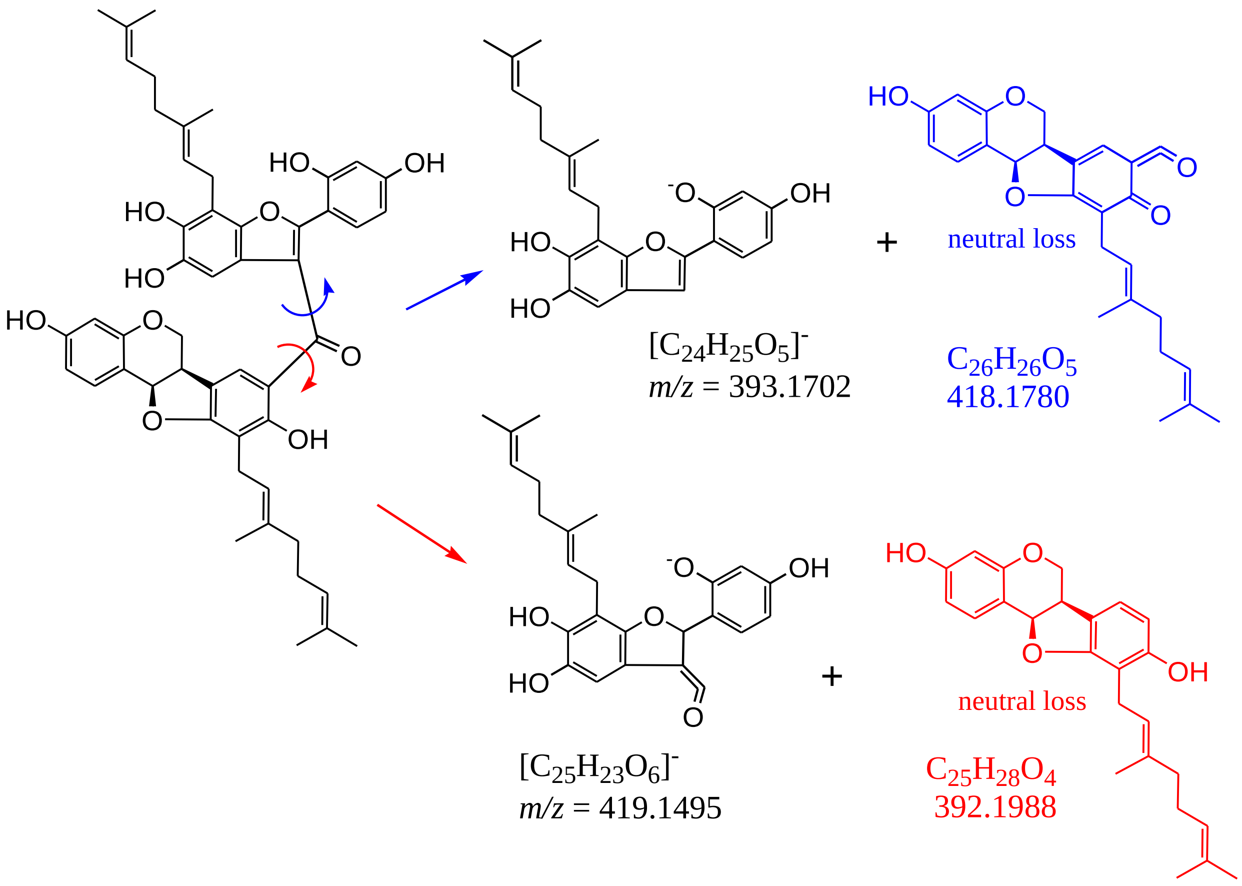

Fig. S12. MS/MS cleavage of the ion [M-H]$^-$ at *m/z* 811.3469 of lespebicolin A (**8**) (negative ion mode)

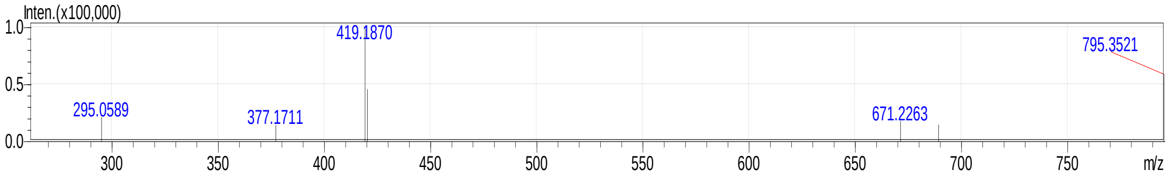

Main frugments:

795.3521 – $[M+H-H_2O]^+$ – $[C_{50}H_{51}O_9]^+$ (Calculated – 795.3528) – H_2O loss

689.2363 – $[M+H- C_9H_{16}]^+$ – $[C_{41}H_{37}O_{10}]^+$ (Calculated – 689.2381) – Geranyl loss

671.2263 – $[M+H-H_2O-C_9H_{16}]^+$ – $[C_{41}H_{35}O_9]^+$ (Calculated – 671.2276) –Geranyl H_2O loss

419.1870 – $[M+H-C_{24}H_{26}O_5]^+$ – $[C_{26}H_{27}O_5]^+$ (Calculated – 419.1853)

377.1711 – $[M+H-C_{26}H_{26}O_5-H_2O]^+$ – $[C_{24}H_{25}O_4]^+$ (Calculated – 377.1747)

295.0579 – $[M+H-C_{24}H_{26}O_5-C_9H_{16}]^+$ – $[C_{17}H_{11}O_5]^+$ (Calculated – 295.0601)

Fig. S13. MS/MS spectrum of the molecular ion at *m/z* 813.3639 of lespebicolin A (**8**) (positive ion mode)

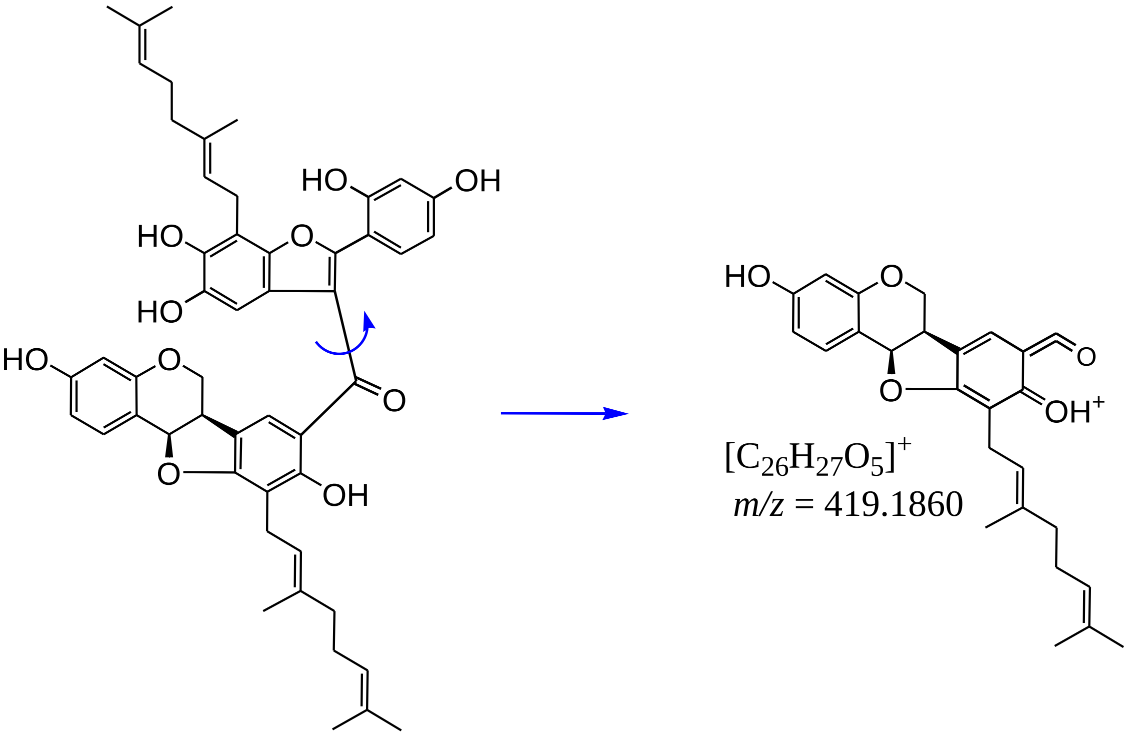

Fig. S14. MS/MS cleavage of the ion $[M+H]^+$ at *m/z* 813.3639 of lespebicolin A (**8**) (positive ion mode)

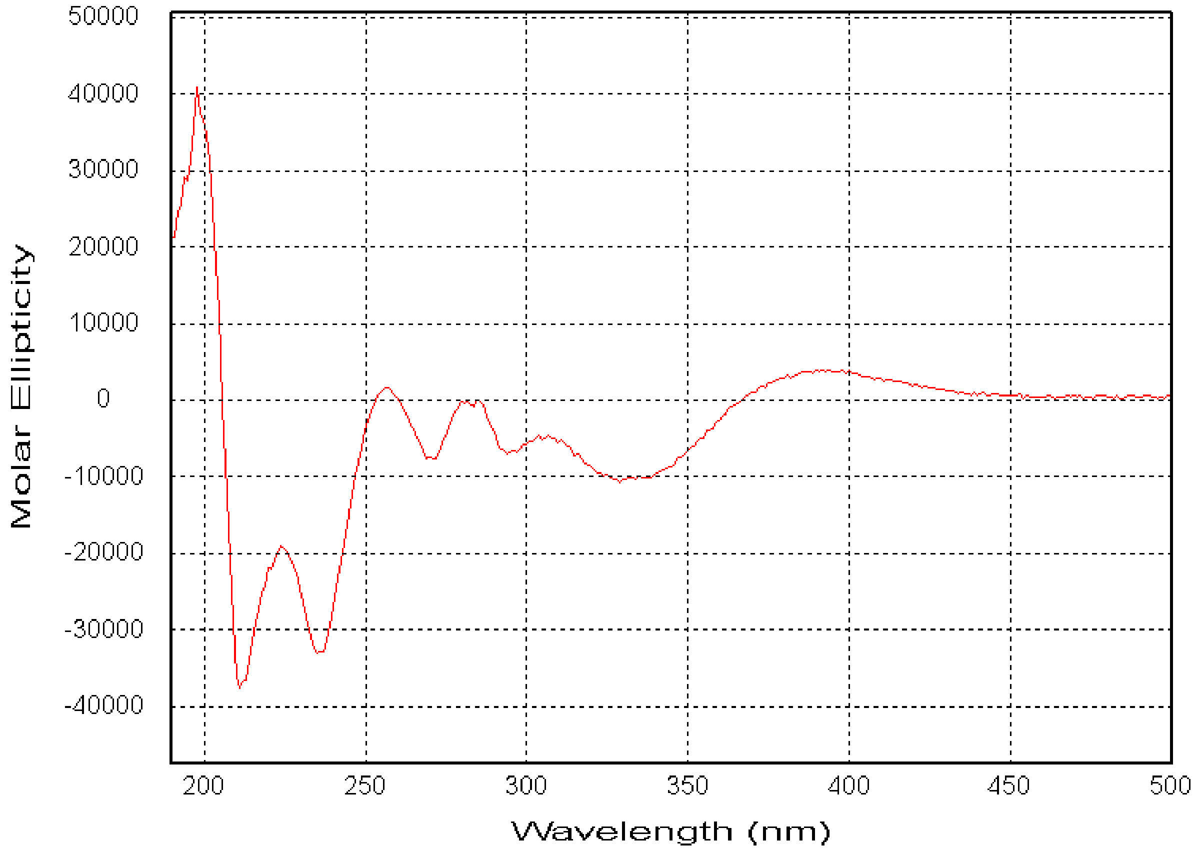

Fig. S15. – CD spectrum of lespebicolin A (**8**)

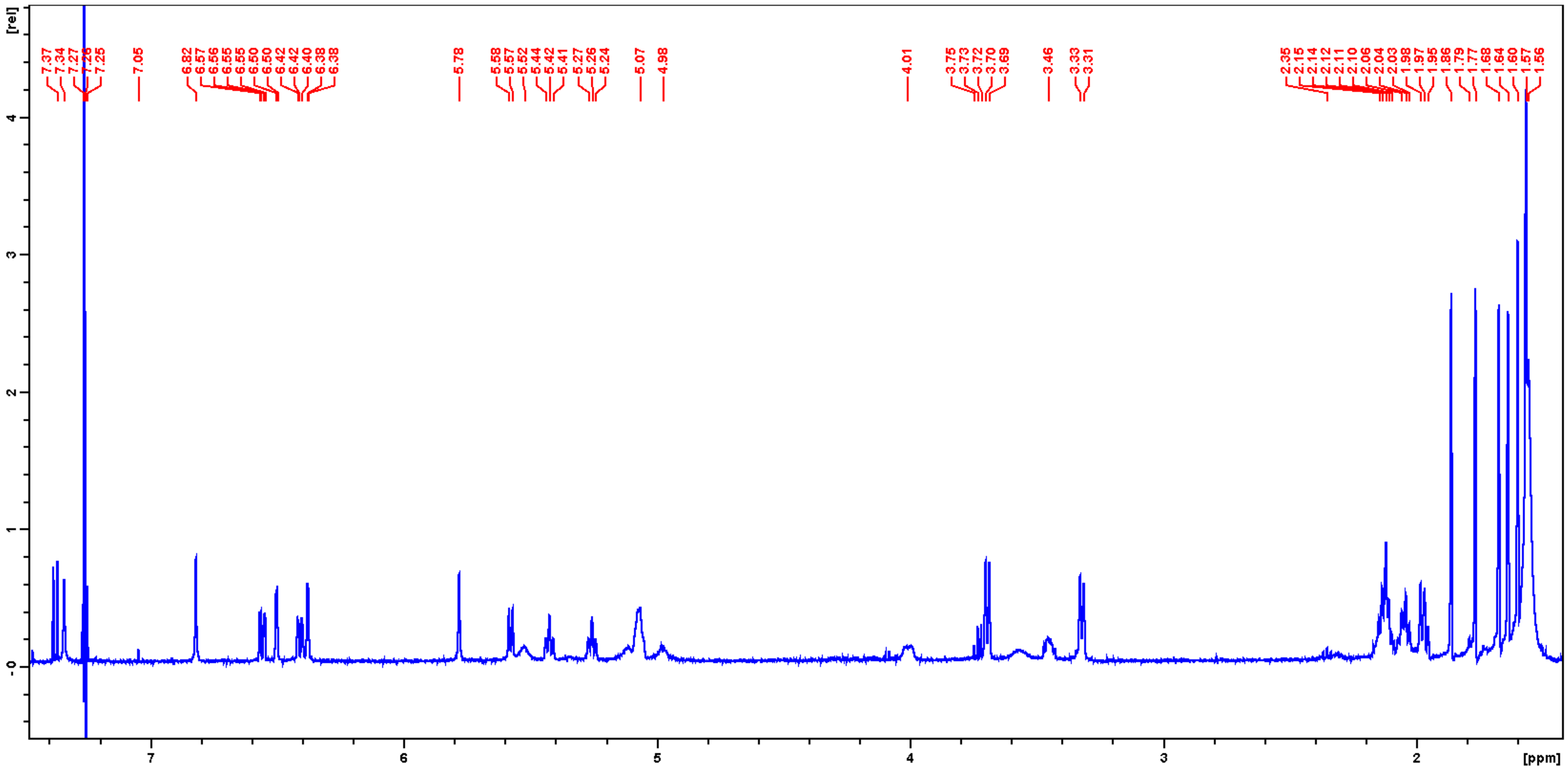

Fig. S16. – ^{1}H spectrum of lespebicolin A (**8**)

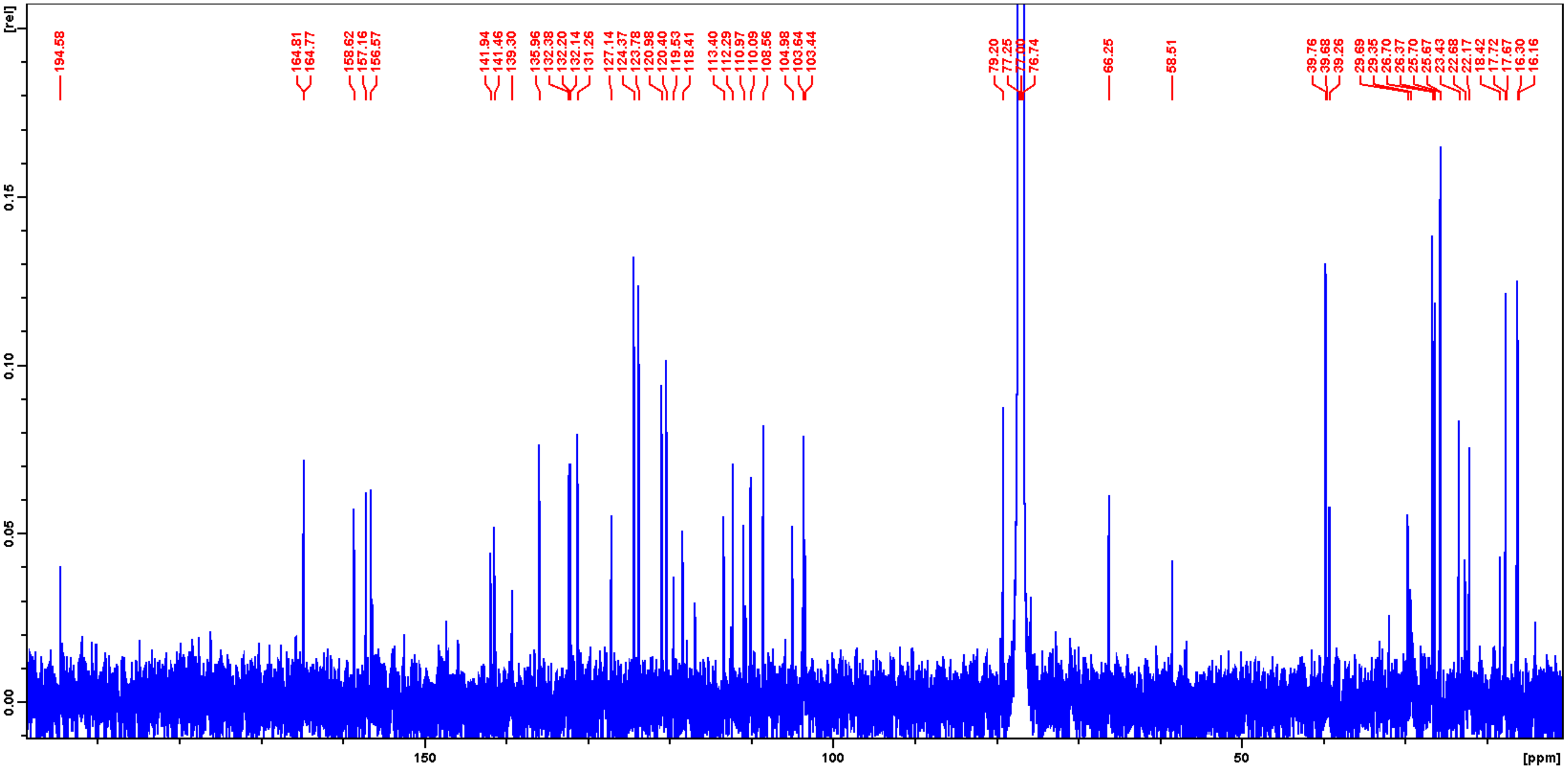

Fig. S17. – ^{13}C spectrum of lespebicolin A (**8**)

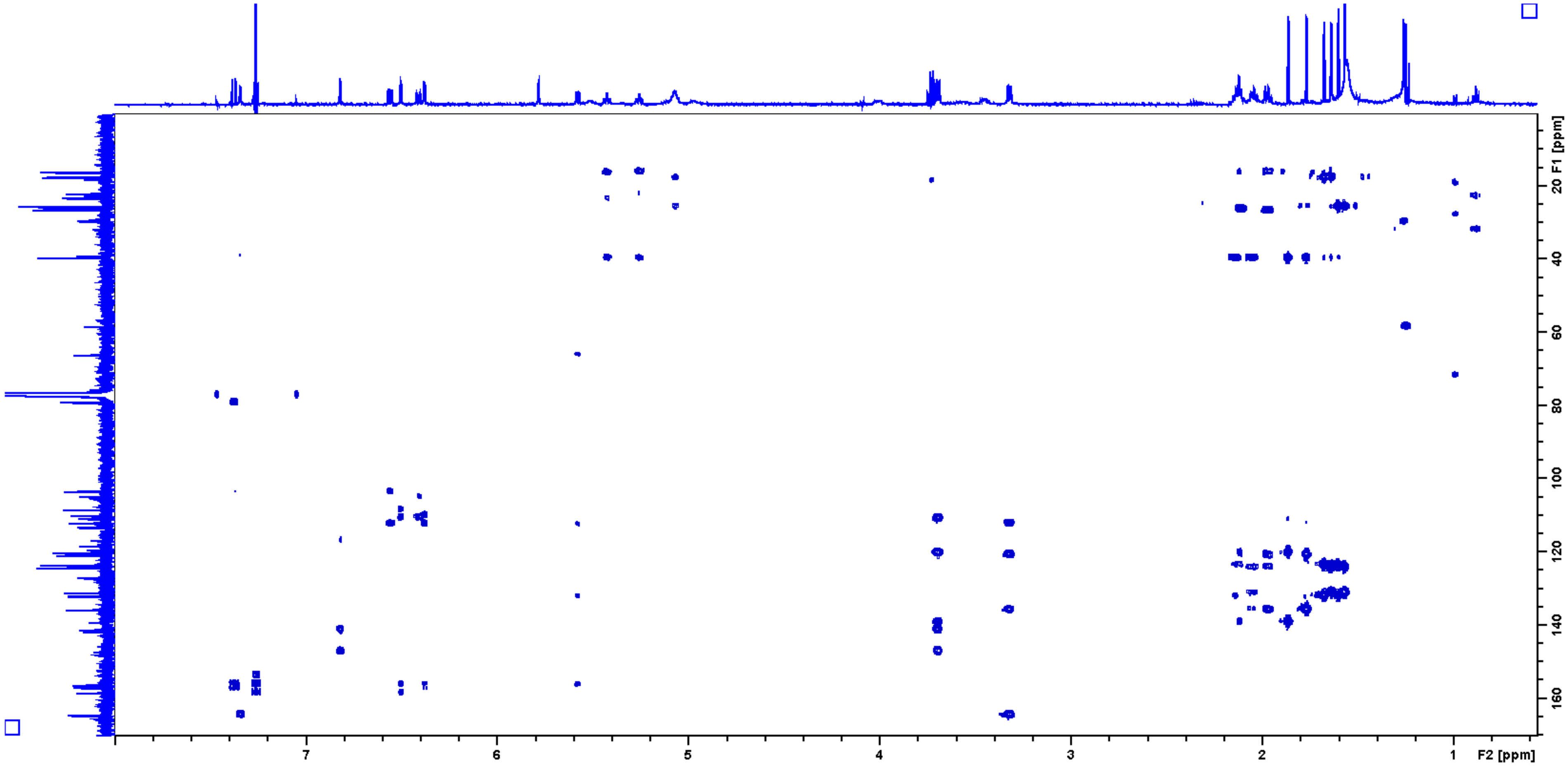

Fig. S18. – HMBC spectrum of lespebicolin A (**8**)

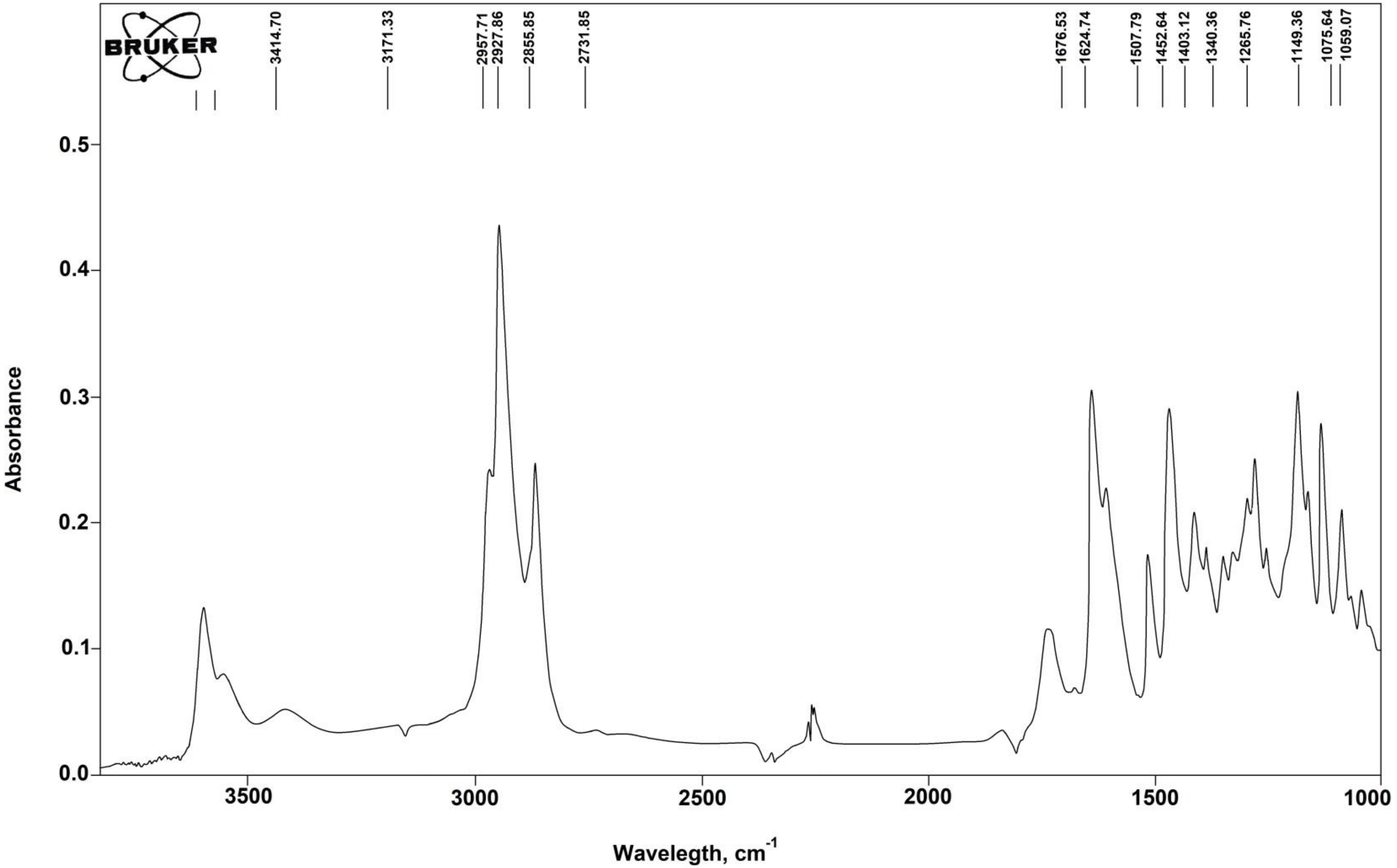

Fig. S19. – IR spectrum of lespebicolin A (**8**)

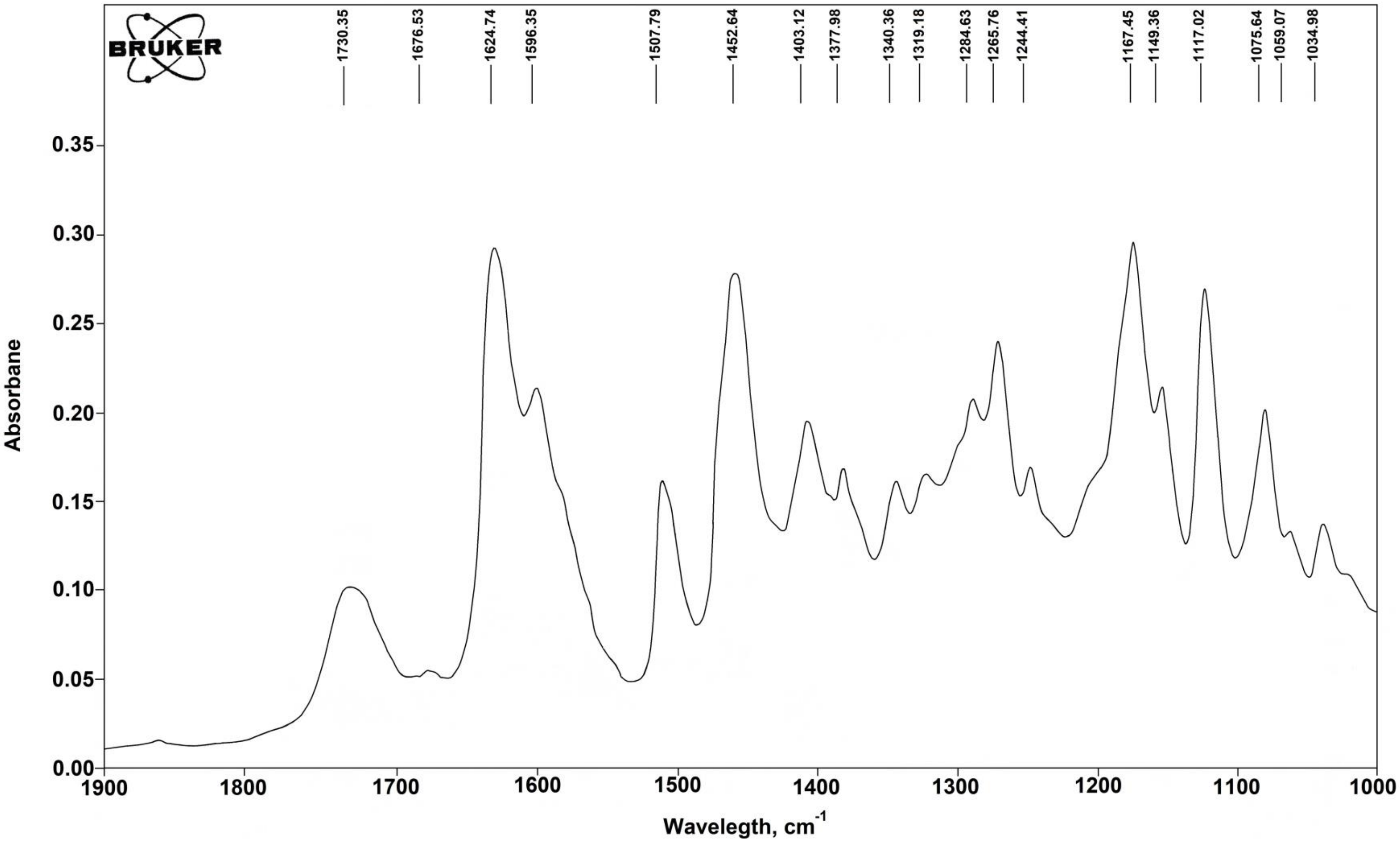

Fig. S20. – IR spectrum of lespebicolin A (**8**)
